# One‐Pot Synthesis of Oxygen Vacancy‐Rich Amorphous/Crystalline Heterophase CaWO_4_ Nanoparticles for Enhanced Radiodynamic‐Immunotherapy

**DOI:** 10.1002/advs.202409551

**Published:** 2024-12-27

**Authors:** Shanshan Peng, Zhen Chen, Jun Wang, Meili Yu, Xuegang Niu, Tingting Cui, Rujiang Ao, Huilan Cai, Hongwei Huang, Lisen Lin, Xiaoyuan Chen, Huanghao Yang

**Affiliations:** ^1^ New Cornerstone Science Laboratory MOE Key Laboratory for Analytical Science of Food Safety and Biology College of Chemistry Fuzhou University Fuzhou 350108 China; ^2^ Department of Neurosurgery Neurosurgery Research Institute the First Affiliated Hospital of Fujian Medical University Fuzhou 350005 China; ^3^ Departments of Diagnostic Radiology, Surgery, Chemical and Biomolecular Engineering and Biomedical Engineering Yong Loo Lin School of Medicine and College of Design and Engineering National University of Singapore Singapore 117597 Singapore; ^4^ Institute of Molecular and Cell Biology 61 Biopolis Drive, Proteos Singapore 138673 Singapore

**Keywords:** CaWO_4_ nanoparticles, enhanced radiodynamic effect, heterophase, oxygen vacancies, radiodynamic‐immunotherapy

## Abstract

Radiodynamic therapy that employs X‐rays to trigger localized reactive oxygen species (ROS) generation can tackle the tissue penetration issue of phototherapy. Although calcium tungstate (CaWO_4_) shows great potential as a radiodynamic agent benefiting from its strong X‐ray absorption and the ability to generate electron–hole (e^−^‐h^+^) pairs, slow charge carrier transfer and fast e^−^‐h^+^ recombination greatly limit its ROS‐generating performance. Herein, via a one‐pot wet‐chemical method, oxygen vacancy‐rich amorphous/crystalline heterophase CaWO_4_ nanoparticles (Ov‐a/c‐CaWO_4_ NPs) with enhanced radiodynamic effect are synthesized for radiodynamic‐immunotherapy of cancer. The phase composition and oxygen vacancy content of CaWO_4_ can be easily tuned by adjusting the solvothermal temperature. More intriguingly, the amorphous/crystalline interfaces and abundant oxygen vacancies accelerate charge carrier transfer and suppress e^−^‐h^+^ recombination, respectively, enabling synergistically improved ROS production from X‐ray‐irradiated Ov‐a/c‐CaWO_4_ NPs. In addition to directly inducing oxidative damage of cancer cells, radiodynamic generation of ROS also boosts immunogenic cell death to provoke a systemic antitumor immune response, thereby allowing the inhibition of both primary and distant tumors as well as cancer metastasis. This study establishes a synergistic enhancement strategy involving the integration of phase and defect engineering to improve the ROS generation capacity of radiodynamic‐immunotherapeutic anticancer nanoagents.

## Graphical abstract

Oxygen vacancy‐rich amorphous/crystalline heterophase CaWO_4_ nanoparticles (Ov‐a/c‐CaWO_4_ NPs) with enhanced radiodynamic effect are synthesized via a one‐pot wet‐chemical approach. The amorphous/crystalline interfaces and oxygen vacancies promote charge carrier transfer and inhibit electron–hole recombination, respectively, allowing synergistically improved reactive oxygen species generation from X‐ray‐irradiated Ov‐a/c‐CaWO_4_ NPs, which elicits immunogenic cell death to enable systemic antitumor immune responses and radiodynamic‐immunotherapy of cancer.

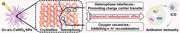

## 1 Introduction

Reactive oxygen species (ROS), which can not only directly cause oxidative damage to cancer cells but also activate systemic antitumor immune responses by triggering immunogenic cell death (ICD), are considered as a potent weapon against cancer.^[^ ^1^ ^]^ As one of the most representative strategies for ROS generation, photodynamic therapy (PDT) relying on light excitation has attracted great attention in precision tumor treatment due to its high therapeutic efficacy and spatiotemporal controllability.^[^ ^2^ ^]^ However, the shallow tissue penetration depth of light seriously hinders its clinical application.^[^ ^3^ ^]^ Intriguingly, X‐rays possess unlimited penetration depth in vivo and have been successfully employed in clinical cancer theranostics including computed tomography imaging and radiotherapy.^[^ ^4^ ^]^ Inspired by this, X‐rays are becoming an alternative excitation source instead of light for initiating the formation of ROS. Given the feasibility of scintillator nanoparticles (NPs) as energy transducers to convert X‐rays into UV–vis light for exciting the adjacent photodynamic agents based on fluorescence resonance energy transfer (FRET), X‐ray‐mediated ROS production can be achieved via indirect activation of PDT process.^[^ ^5^ ^]^ Despite that X‐ray‐activated PDT (X‐PDT) offers desirable penetration depth, highly efficient FRET systems often suffer from elaborate design and complicated synthesis since FRET efficiency is strongly dependent on the donor‐acceptor distance and spectral overlap integral.^[^ ^6^ ^]^ From this perspective, radiodynamic therapy (RDT) that employs ROS‐generating radiodynamic agents with the ability to be excited directly by X‐rays may represent a superior therapeutic strategy than conventional X‐PDT.

Radiodynamic agents as the indispensable component of RDT always play a critical role in directly responding to X‐rays to elicit ROS generation.^[^ ^7^ ^]^ Recently, high‐Z element‐contained semiconductor nanomaterials which may yield electron–hole (e^−^‐h^+^) pairs under X‐ray irradiation, similar to photocatalysts capable of producing ROS by photogenerated e^−^‐h^+^ pairs, have become potential radiodynamic agents.^[^ ^8^ ^]^ In particular, calcium tungstate (CaWO_4_) NPs are expected to be a fascinating candidate for RDT because of their strong X‐ray absorption and the capacity to generate e^−^‐h^+^ pairs.^[^ ^9^ ^]^ Nevertheless, traditional CaWO_4_ NPs often exhibit poor ROS‐generating efficiency resulting from slow charge carrier transfer and fast e^−^‐h^+^ recombination, which greatly limits their radiodynamic efficacy. There are several ways to improve the ROS‐generating properties of semiconductor nanomaterials, such as phase engineering and defect engineering.^[^ ^10^ ^]^ On the one hand, phase engineering, especially the construction of amorphous/crystalline heterophase structures, is an effective approach to boost charge carrier transfer through introducing heterophase interfaces.^[^ ^11^ ^]^ On the other hand, defect engineering, particularly the creation of vacancy defects, is a common method to suppress e^−^‐h^+^ recombination via providing charge traps.^[^ ^12^ ^]^ Although promising for synergistic enhancement of ROS‐generating capability, the combination of phase and defect engineering toward CaWO_4_‐based radiodynamic agents has not yet been reported.

**Scheme 1:**
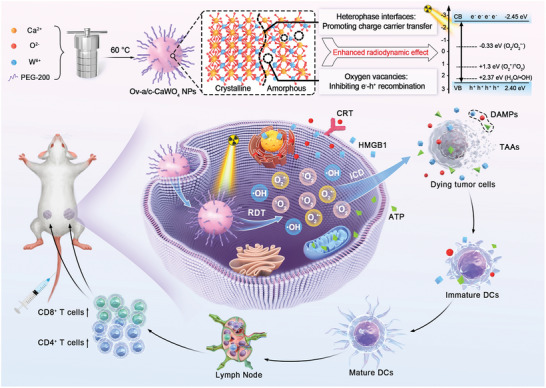
Schematic representation of the one‐pot solvothermal synthesis of Ov‐a/c‐CaWO_4_ NPs with enhanced radiodynamic effect for low‐dose X‐ray‐triggered radiodynamic‐immunotherapy of cancer.

Herein, we present the one‐pot wet‐chemical synthesis of oxygen vacancy‐rich amorphous/crystalline heterophase CaWO_4_ nanoparticles (Ov‐a/c‐CaWO_4_ NPs) for enhanced radiodynamic‐immunotherapy of cancer (**Scheme** **1**). The phase composition and oxygen vacancy defects of CaWO_4_‐based radiodynamic nanoagents can be easily controlled by regulating the solvothermal temperature. Importantly, the optimized Ov‐a/c‐CaWO_4_ NPs prepared at 60 °C had a superior radiodynamic effect for ROS generation under low‐dose X‐ray irradiation (0.5 Gy), which could be attributed to the fact that their amorphous/crystalline interfaces and abundant oxygen vacancies strongly promoted charge carrier transfer and inhibited e^−^‐h^+^ recombination, respectively, allowing highly efficient X‐ray‐triggered ROS production. In addition to directly destroying cancer cells, the radiodynamically generated ROS by X‐ray‐irradiated Ov‐a/c‐CaWO_4_ NPs also elicited robust ICD with the release of damage‐associated molecular patterns (DAMPs), which enabled a systemic antitumor immune response and subsequent effective radiodynamic‐immunotherapy of cancer. This study offers a versatile strategy to synergistically improve the ROS generation capacity of radiodynamic‐immunotherapeutic anticancer nanoagents through integrating the phase and defect engineering.

## 2 Results and Discussion

### 2.1 Fabrication and Characterization of Ov‐a/c‐CaWO_4_ NPs

**Figure 1:**
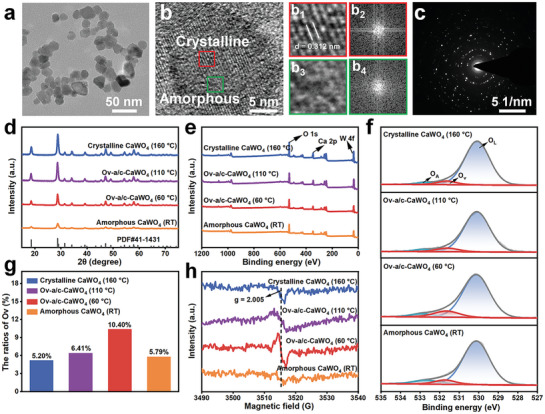
Characterization of Ov‐a/c‐CaWO_4_ NPs. a) TEM and b) high‐resolution TEM images of Ov‐a/c‐CaWO_4_ NPs. b_1_) The representative crystalline domain marked by the red square in (b) and b_2_) its fast Fourier transform (FFT) pattern. b_3_) The representative amorphous domain marked by the green square in (b) and b_4_) its FFT pattern. c) SAED image of Ov‐a/c‐CaWO_4_ NPs. d) XRD patterns of CaWO_4_ NPs prepared at different temperatures including RT, 60 °C, 110 °C, or 160 °C. e) Survey and f) O 1s XPS spectra of CaWO_4_ NPs prepared at different temperatures. O_L_: lattice oxygen, O_V_: oxygen vacancies, O_A_: surface‐adsorbed oxygen species. g) The ratios of Ov in O 1s XPS spectra. h) EPR spectra of CaWO_4_ NPs prepared at various temperatures.

The Ov‐a/c‐CaWO_4_ NPs with amorphous/crystalline heterophase interfaces and rich oxygen vacancies were prepared via a one‐pot solvothermal process at 60 °C using polyethylene glycol 200 (PEG‐200) as the solvent. As can be seen in the transmission electron microscopy (TEM) images (**Figures** **1**a,b and S1 and S2, Supporting Information), Ov‐a/c‐CaWO_4_ NPs were composed of amorphous and crystalline domains. Moreover, the selected area electron diffraction (SAED) pattern displayed weak halo diffraction with some distinct dots, further suggesting the coexistence of amorphous and crystalline phases in Ov‐a/c‐CaWO_4_ NPs (Figure 1c). Intriguingly, the phase composition of CaWO_4_ NPs could be tuned by varying the solvothermal temperature. The proportion of amorphous to crystalline component decreased with increasing temperature, as evidenced by the TEM and X‐ray diffraction (XRD) measurements (Figures 1d and S3 and S4, Supporting Information). Thus, through changing the synthetic temperature from room temperature (RT) to 160 °C, a series of CaWO_4_ NPs with different crystallinities including amorphous, amorphous/crystalline, and crystalline phases could be obtained.

In order to examine the oxygen vacancy defects of Ov‐a/c‐CaWO_4_ NPs, X‐ray photoelectron spectroscopy (XPS) measurements were carried out. As can be seen in the XPS spectra (Figures 1e,f and S5, Supporting Information), the O 1s spectra of CaWO_4_ NPs synthesized at various temperatures could be divided into three peaks at about 530.1, 531.6, and 532.8 eV, corresponding to lattice oxygen, oxygen vacancies, and surface‐adsorbed oxygen species, respectively. Furthermore, the ratios of oxygen vacancies in CaWO_4_ NPs prepared at RT, 60, 110, or 160 °C were calculated to be 5.79%, 10.40%, 6.41%, or 5.20%, respectively (Figure 1g). The oxygen vacancy content increased with decreasing solvothermal temperature from 160 to 60 °C because the crystallinity decreased gradually. In addition, the low oxygen vacancy content of amorphous CaWO_4_ NPs prepared at RT was probably due to the fact that high content of adsorbed oxygen species in amorphous materials could provide oxygen atoms for the self‐healing of oxygen vacancies.^[^ ^13^ ^]^ An evident electron paramagnetic resonance (EPR) signal at g = 2.005 was detected for Ov‐a/c‐CaWO_4_ NPs synthesized at 60 °C (Figure 1h), which also confirmed the presence of abundant oxygen vacancies.^[^ ^14^ ^]^ The above results indicated that simultaneous phase and defect engineering of CaWO_4_‐based radiodynamic nanoagents via a one‐pot solvothermal route endows them with both amorphous/crystalline heterophase structure and oxygen vacancies.

### 2.2 Radiodynamic Performance and Mechanism of Ov‐a/c‐CaWO_4_ NPs

**Figure 2:**
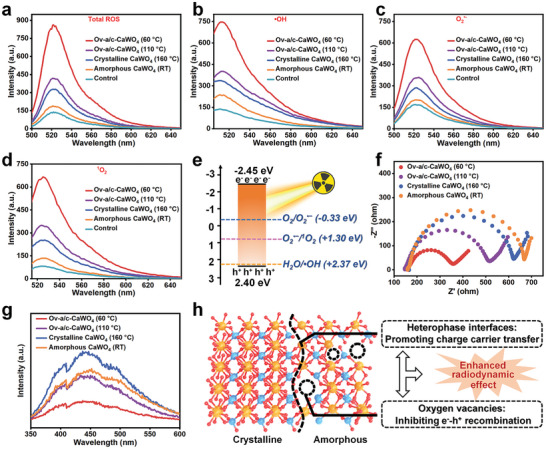
Radiodynamic ROS generation performance and mechanism of Ov‐a/c‐CaWO_4_ NPs. a) Fluorescence spectra of 2′,7′‐dichlorodihydrofluorescein (DCFH) mixed with various CaWO_4_ NPs synthesized at different temperatures (including RT, 60, 110, or 160 °C) after X‐ray irradiation (0.5 Gy). The generation of b) •OH, c) O_2_^•−^, and d) ^1^O_2_ from diverse CaWO_4_ NPs under X‐ray irradiation at 0.5 Gy, measured by aminophenyl fluorescein (APF), dihydrorhodamine 123 (DHR 123), and singlet oxygen sensor green (SOSG), respectively. e) The band structures of Ov‐a/c‐CaWO_4_ NPs and the energy levels of •OH‐, O_2_^•−^‐, and ^1^O_2_‐generating processes. f) EIS and g) RL spectra of CaWO_4_ NPs prepared at different temperatures. h) Schematic illustration of the synergistic enhancement mechanism of amorphous/crystalline heterophase interfaces and oxygen vacancies on the radiodynamic effect of Ov‐a/c‐CaWO_4_ NPs.

The enhanced radiodynamic ROS generation of Ov‐a/c‐CaWO_4_ NPs was evaluated using 2′,7′‐dichlorodihydrofluorescein (DCFH) as a fluorescent probe. As presented in **Figures** **2**a and S6, Supporting Information, under low‐dose X‐ray irradiation (0.5 Gy), the Ov‐a/c‐CaWO_4_ NPs prepared at 60 °C exhibited a significantly higher ROS‐generating activity than both the amorphous and crystalline CaWO_4_ NPs. It can be seen in Figures 2b–d and S7 (Supporting Information), that the main types of ROS formed by X‐ray‐irradiated Ov‐a/c‐CaWO_4_ NPs were hydroxyl radicals (•OH), superoxide radicals (O_2_^•−^), and singlet oxygen (^1^O_2_), which we attribute to their strong X‐ray absorption and capacity of producing e^−^‐h^+^ pairs. The X‐ray‐generated holes can react with water (H_2_O) molecules to yield •OH, and the electrons can transform oxygen (O_2_) into O_2_^•−^ that will be further oxidized to ^1^O_2_ by holes.^[^ ^15^ ^]^ In addition, the PEG‐200‐coated Ov‐a/c‐CaWO_4_ NPs showed good colloidal and X‐ray stability (Figures S8–S10, Supporting Information), thereby providing support for RDT applications.

To elucidate the radiodynamic mechanism of Ov‐a/c‐CaWO_4_ NPs to generate diverse ROS, the energy band structure was studied. The bandgap of Ov‐a/c‐CaWO_4_ NPs synthesized at 60 °C was estimated to be 4.85 eV by the Kubelka−Munk formula from the UV–vis diffuse reflectance spectrum (DRS; Figure S11, Supporting Information). Combining with the valence band XPS (VB‐XPS) results, the valence band (VB) and conduction band (CB) potentials of Ov‐a/c‐CaWO_4_ NPs were 2.40 and −2.45 eV, respectively (Figure S12, Supporting Information), which are matched with the energy levels of the •OH‐, O_2_^•−^‐, and ^1^O_2_‐generating processes (Figure 2e). Next, electrochemical impedance spectroscopy (EIS) was applied to assess the charge transfer efficiency. As shown in Figure 2f, the semicircle in the EIS plots of Ov‐a/c‐CaWO_4_ NPs prepared at 60 °C was much smaller than that of amorphous and crystalline CaWO_4_ NPs, implying the reduced charge transfer resistance in Ov‐a/c‐CaWO_4_ NPs with amorphous/crystalline heterophase structure, which could be ascribed to the fact that heterophase interfaces are able to accelerate charge carrier transfer. Furthermore, radioluminescence (RL) analysis was conducted to investigate the recombination rate of X‐ray‐generated e^−^‐h^+^ pairs.^[^ ^10^, ^16^ ^]^ As expected, Ov‐a/c‐CaWO_4_ NPs with abundant oxygen vacancies showed weaker RL intensity compared to amorphous and crystalline CaWO_4_ NPs (Figure 2g), indicating the efficient suppression of e^−^‐h^+^ recombination in Ov‐a/c‐CaWO_4_ NPs. Additionally, the RL intensity of CaWO_4_ NPs was reduced with increasing oxygen vacancy content. Therefore, it can be deduced that oxygen vacancies serve as charge traps to inhibit the radiative recombination of X‐ray‐generated e^−^‐h^+^ pairs during the radiodynamic process. These findings demonstrated that the amorphous/crystalline heterophase structure and oxygen vacancies of Ov‐a/c‐CaWO_4_ NPs could synergistically enhance their radiodynamic effect by facilitating charge carrier transfer and suppressing e^−^‐h^+^ recombination (Figure 2h).

### 2.3 Radiodynamic Efficacy of Ov‐a/c‐CaWO_4_ NPs In Vitro

**Figure 3:**
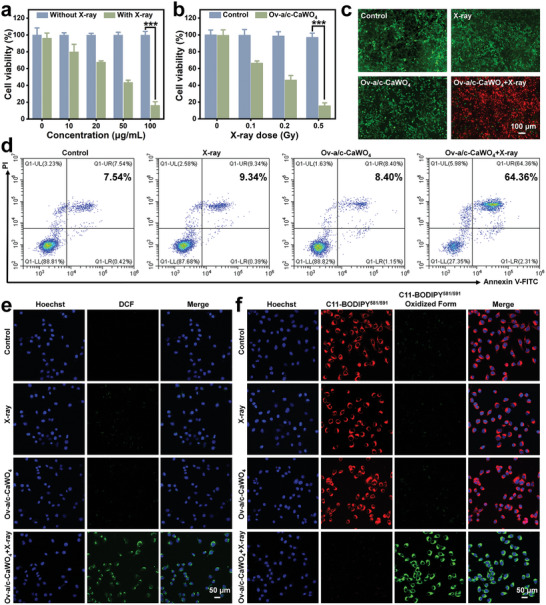
In vitro RDT performance of Ov‐a/c‐CaWO_4_ NPs. a) Cell viability of 4T1 cells exposed to different concentrations of Ov‐a/c‐CaWO_4_ NPs for 12 h and then treated with or without X‐ray irradiation (0.5 Gy). n = 3. b) Cell viability of Ov‐a/c‐CaWO_4_ NPs‐incubated 4T1 cells after exposure to X‐ray irradiation at various doses ([Ov‐a/c‐CaWO_4_ NPs] = 100 µg mL^−1^). n = 3. c) Calcein‐AM/PI costaining of 4T1 cells treated with X‐ray irradiation, Ov‐a/c‐CaWO_4_ NPs, or Ov‐a/c‐CaWO_4_ NPs plus X‐ray irradiation. d) Flow cytometry analysis of cancer cell apoptosis after diverse treatments. Fluorescence images of 4T1 cells stained with e) DCFH‐DA or f) C11‐BODIPY^581/591^ after treatment with X‐ray irradiation, Ov‐a/c‐CaWO_4_ NPs, or Ov‐a/c‐CaWO_4_ NPs plus X‐ray irradiation (0.5 Gy). Data are presented as mean ± SD. ^***^ *p* <0.001.

Considering their superior ROS production ability under low‐dose X‐ray irradiation, Ov‐a/c‐CaWO_4_ NPs synthesized at 60 °C were selected for the following radiodynamic‐immunotherapy studies. Prior to assessing the in vitro RDT efficacy, the cellular internalization of Ov‐a/c‐CaWO_4_ NPs was first determined. As imaged by confocal laser scanning microscopy, the fluorescence intensity of 4T1 tumor cells increased in a time‐dependent manner after incubation with cyanine 5.5 (Cy5.5)‐labeled Ov‐a/c‐CaWO_4_ NPs (Figure S13, Supporting Information), suggesting the effective cellular internalization of Ov‐a/c‐CaWO_4_ NPs. Following this, cell count kit‐8 (CCK‐8) assay was performed to evaluate the radiodynamic cytotoxicity of Ov‐a/c‐CaWO_4_ NPs against tumor cells. As presented in **Figure** **3**a, when exposed to a low‐dose of X‐ray irradiation (0.5 Gy), the viability of 4T1 cells decreased significantly with increasing concentration of Ov‐a/c‐CaWO_4_ NPs. In contrast, neither Ov‐a/c‐CaWO_4_ NPs nor 0.5 Gy X‐ray alone caused obvious cancer cell death (Figure S14, Supporting Information). Meanwhile, Ov‐a/c‐CaWO_4_ NPs exerted a noticeable tumor cell‐killing effect even at an X‐ray irradiation dose as low as 0.1 Gy (Figure 3b), which is benefited from their enhanced radiodynamic ROS generation. To intuitively investigate the radiodynamic anticancer effect of Ov‐a/c‐CaWO_4_ NPs, a live/dead cell staining assay based on calcein‐AM/propidium iodide (PI) was conducted. As expected, Ov‐a/c‐CaWO_4_ NPs effectively elicited cancer cell death under low‐dose X‐ray irradiation (Figure 3c), which was also supported by apoptotic analysis using flow cytometry (Figure 3d). Then, intracellular ROS generation by Ov‐a/c‐CaWO_4_ NPs upon X‐ray irradiation was further validated with 2′,7′‐dichlorodihydrofluorescein diacetate (DCFH‐DA) fluorescent probe. As displayed in Figures 3e and S15 (Supporting Information), a strong green fluorescence was observed in 4T1 cells incubated with Ov‐a/c‐CaWO_4_ NPs followed by X‐ray irradiation, while negligible signal was detected in cells treated with Ov‐a/c‐CaWO_4_ NPs or X‐ray irradiation alone, thereby proving the efficient radiodynamic ROS formation by Ov‐a/c‐CaWO_4_ NPs inside cancer cells. Moreover, Ov‐a/c‐CaWO_4_ NPs‐mediated radiodynamic production of ROS led to lipid peroxidation (LPO) and lysosomal damage (Figures 3f and S16, Supporting Information). Collectively, these results demonstrated the potential of Ov‐a/c‐CaWO_4_ NPs as a potent RDT agent to achieve remarkable anticancer therapy under low‐dose X‐ray irradiation.

### 2.4 In Vitro Immune Activation by X‐Ray‐Irradiated Ov‐a/c‐CaWO_4_ NPs

**Figure 4:**
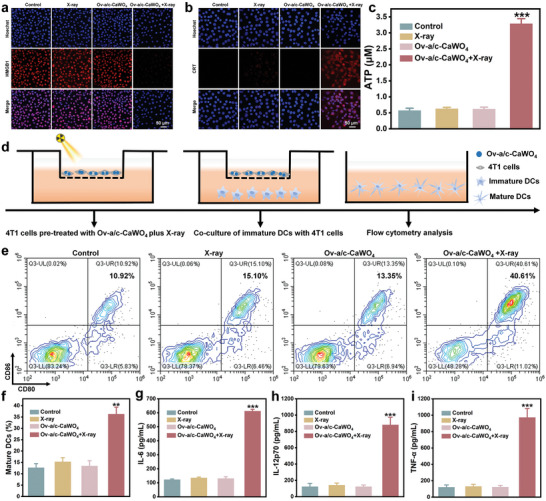
Radiodynamically generated ROS‐triggered ICD and immune response by Ov‐a/c‐CaWO_4_ NPs in vitro. Immunofluorescence staining of a) HMGB1 release and b) CRT exposure in 4T1 cells treated with X‐ray irradiation, Ov‐a/c‐CaWO_4_ NPs, or Ov‐a/c‐CaWO_4_ NPs plus X‐ray irradiation. c) The levels of released ATP in cell culture medium from different groups. n = 3. d) Schematic illustration of co‐culture system established for evaluating the maturation of DCs after exposure to 4T1 cells pre‐treated with Ov‐a/c‐CaWO_4_ NPs plus X‐ray irradiation (0.5 Gy). e) Flow cytometry and f) quantitative analysis of CD80^+^CD86^+^ DCs after exposure to 4T1 cells with different treatments (gated on CD11c^+^ cells). n = 3. The secretion of g) IL‐6, h) IL‐12p70, and i) TNF‐α in cell culture medium from various groups. n = 3. Data are presented as mean ± SD. ^**^ *p* <0.01, ^***^ *p* <0.001.

Except for the direct tumoricidal effect of X‐ray‐irradiated Ov‐a/c‐CaWO_4_ NPs via ROS‐triggered oxidative damage toward cancer cells, their ability to radiodynamically initiate ICD‐induced immune activation was also examined in vitro. Traditionally, the immunogenicity of ROS‐elicited ICD is mainly mediated by DAMPs, including high‐mobility group box 1 (HMGB1), calreticulin (CRT), and adenosine triphosphate (ATP).^[^ ^17^ ^]^ As presented in **Figures** **4**a and S17 (Supporting Information), the HMGB1 level in the nuclei of Ov‐a/c‐CaWO_4_ NPs‐incubated 4T1 cells was significantly reduced after low‐dose X‐ray irradiation (0.5 Gy). Meanwhile, CRT exposure on the membrane surface of 4T1 cells treated with Ov‐a/c‐CaWO_4_ NPs plus X‐ray irradiation showed an apparent increase compared with control groups (Figures 4b and S18, Supporting Information). Besides, a markedly elevated ATP release was observed when the Ov‐a/c‐CaWO_4_ NPs‐incubated cells were exposed to X‐ray irradiation at 0.5 Gy (Figure 4c). Therefore, these results confirmed the feasibility of using Ov‐a/c‐CaWO_4_ NPs with superior radiodynamic effect for ICD induction.

During the ICD of tumor cells, dendritic cells (DCs) as the main antigen‐presenting cells can capture and process tumor‐associated antigens (TAAs), accompanied by the maturation of DCs.^[^ ^18^ ^]^ The transition from immature to mature DCs was preliminarily explored by establishing a co‐culture system composed of DCs and 4T1 cells (Figure 4d). Flow cytometry analysis verified that the proportion of mature DCs (CD80^+^CD86^+^ DCs) was substantially increased in the group exposed to Ov‐a/c‐CaWO_4_ NPs plus X‐ray irradiation (0.5 Gy), indicating the capacity of Ov‐a/c‐CaWO_4_ NPs to induce the maturation of DCs with the assistance of low‐dose X‐ray (Figure 4e,f). Importantly, mature DCs secrete several pro‐inflammatory cytokines to promote T cell‐mediated immune responses.^[^ ^19^ ^]^ The secretion of pro‐inflammatory cytokines including interleukin‐6 (IL‐6), interleukin‐12p70 (IL‐12p70), and tumor necrosis factor‐α (TNF‐α) was measured by enzyme‐linked immunosorbent assay (ELISA). As shown in Figures 4g–i and S19 (Supporting Information), the secretion of these cytokines from DCs was dramatically enhanced after co‐culture with 4T1 cells pre‐subjected to Ov‐a/c‐CaWO_4_ NPs incubation plus X‐ray irradiation. Overall, the above results supported the validity of Ov‐a/c‐CaWO_4_ NPs for RDT‐triggered immune response.

### 2.5 In Vivo Radiodynamic‐Immunotherapy with Ov‐a/c‐CaWO_4_ NPs

**Figure 5:**
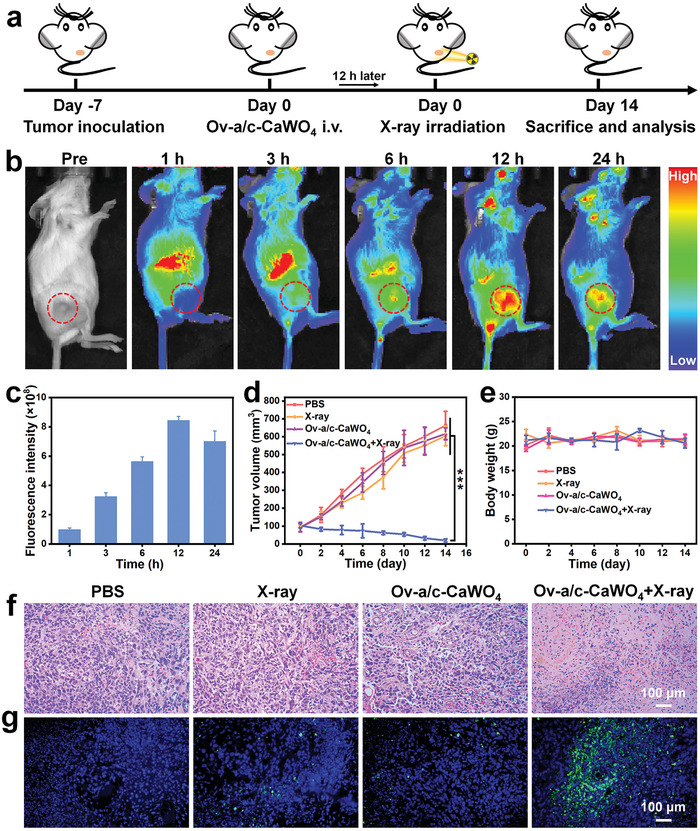
In vivo tumor accumulation and X‐ray‐triggered antitumor efficacy of Ov‐a/c‐CaWO_4_ NPs. a) Therapeutic schedule for 4T1 tumor‐bearing mice. b) Fluorescence images of 4T1 tumor‐bearing mice and c) the corresponding fluorescence intensity in tumors after i.v. injection of Cy5.5‐labeled Ov‐a/c‐CaWO_4_ NPs. n = 3. d) Tumor growth curves of 4T1 tumor‐bearing mice after treatment with PBS, X‐ray irradiation, Ov‐a/c‐CaWO_4_ NPs, or Ov‐a/c‐CaWO_4_ NPs plus X‐ray irradiation. n = 6. e) Body weight curves of different groups of mice. n = 6. f) H&E and g) TUNEL staining of tumor sections harvested from various groups. Data are presented as mean ± SD. ^***^ *p* <0.001.

Encouraged by their potent radiodynamic cytotoxicity against cancer cells and immune activation ability, the in vivo antitumor efficacy of Ov‐a/c‐CaWO_4_ NPs was assessed on 4T1 tumor‐bearing mice model (**Figure** **5**a). The in vivo tumor accumulation was first monitored by fluorescence imaging upon intravenous (i.v.) injection of Cy5.5‐labeled Ov‐a/c‐CaWO_4_ NPs. It can be seen in the fluorescence images that the fluorescence intensity in tumor region enhanced gradually and remained high for up to 24 h post‐injection (Figures 5b,c and S20, Supporting Information), implying efficient accumulation of Ov‐a/c‐CaWO_4_ NPs in tumor. Then, the tumor growth inhibition was evaluated after i.v. administration of Ov‐a/c‐CaWO_4_ NPs, followed by low‐dose of X‐ray irradiation (0.5 Gy) at 12 h post‐injection. Notably, tumor growth in mice treated with Ov‐a/c‐CaWO_4_ NPs plus X‐ray irradiation was greatly suppressed (Figure 5d), resulting from the superior radiodynamic effect and effective tumor uptake of Ov‐a/c‐CaWO_4_ NPs. Moreover, neither notable body weight loss nor distinct histological abnormality in major organs was found during the therapeutic process (Figures 5e and S21–S23, Supporting Information). The antitumor efficacy was also verified by hematoxylin and eosin (H&E) staining. As presented in Figure 5f, tumor tissues in mice injected intravenously with Ov‐a/c‐CaWO_4_ NPs and subsequently exposed to X‐ray irradiation suffered more serious damage compared to those in mice treated with Ov‐a/c‐CaWO_4_ NPs or X‐ray irradiation alone, indicating the potent therapeutic effect of X‐ray‐irradiated Ov‐a/c‐CaWO_4_ NPs. In addition, a higher level of cellular apoptosis was observed in tumors harvested from mice treated with Ov‐a/c‐CaWO_4_ NPs plus X‐ray irradiation (Figure 5g), as validated by the terminal deoxynucleotidyl transferase‐mediated dUTP nick‐end labeling (TUNEL) assay. These findings suggested that Ov‐a/c‐CaWO_4_ NPs could serve as a promising radiodynamic agent for tumor treatment.

**Figure 6:**
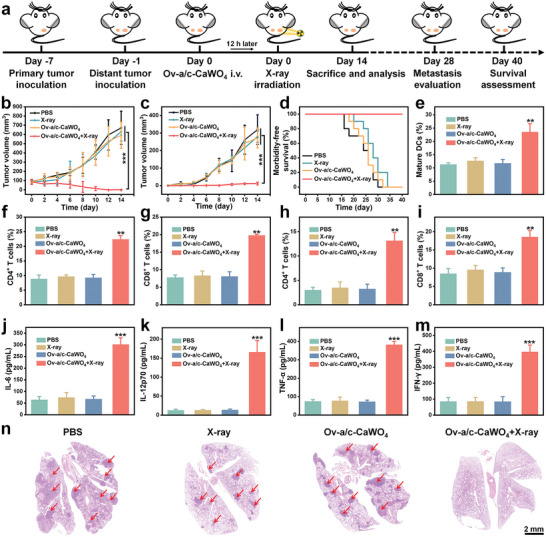
Tumor metastasis inhibition by Ov‐a/c‐CaWO_4_ NPs‐mediated radiodynamic‐immunotherapy. a) Therapeutic schedule for bilateral 4T1 tumor model. Tumor growth curves of b) primary and c) distant tumors in bilateral 4T1 tumor‐bearing mice after treatment with PBS, X‐ray irradiation, Ov‐a/c‐CaWO_4_ NPs, or Ov‐a/c‐CaWO_4_ NPs plus X‐ray irradiation (0.5 Gy). n = 6. d) Survival curves of mice after different treatments. n = 10. e) Quantitative analysis by flow cytometry of mature DCs (CD80^+^CD86^+^ DCs) in tumor‐draining lymph nodes of mice after various treatments (gated on CD11c^+^ cells). n = 3. Quantitative analysis by flow cytometry of CD4^+^ T cells (CD3^+^CD4^+^) and CD8^+^ T cells (CD3^+^CD8^+^) in f, g) spleens or h, i) distant tumors of different groups of mice (gated on CD3^+^ cells). n = 3. The levels of j) IL‐6, k) IL‐12p70, l) TNF‐α, and m) IFN‐γ in the serum of mice from various groups. n = 3. n) H&E staining of lung tissues harvested from different groups on day 28. Red arrows indicate the metastatic nodules. Data are presented as mean ± SD. ^**^ *p* <0.01, ^***^ *p* <0.001.

To investigate whether radiodynamic ROS generation by Ov‐a/c‐CaWO_4_ NPs can elicit tumor immunogenicity for suppressing tumor metastasis, a bilateral 4T1 tumor model was established (**Figure** **6**a). When primary tumor size reached about 80 mm^3^, the mice were inoculated with distant tumors to artificially simulate tumor metastasis and then subjected to i.v. injection of Ov‐a/c‐CaWO_4_ NPs as well as X‐ray irradiation of primary tumors at 12 h post‐injection. As shown in Figures 6b–d and S24–S26 (Supporting Information), the treatment of Ov‐a/c‐CaWO_4_ NPs plus X‐ray irradiation displayed substantial growth inhibition of both primary and distant tumors, suggesting that X‐ray‐activated Ov‐a/c‐CaWO_4_ NPs could trigger a robust systemic antitumor immune response to achieve abscopal therapeutic effect. To further clarify the immunotherapeutic mechanisms, immune cells in the lymph node, spleen, and tumor as well as immunostimulatory cytokines in the serum were analyzed at day 14 after treatment. First, we assessed the maturation status of DCs in tumor‐draining lymph nodes using flow cytometry. Similar to the in vitro results, Ov‐a/c‐CaWO_4_ NPs plus X‐ray irradiation notably promoted the maturation of DCs (Figures 6e and S27, Supporting Information), which would subsequently evoke T‐cell immunity. As anticipated, splenic‐activated CD4^+^ and CD8^+^ T cells were significantly increased in mice treated with Ov‐a/c‐CaWO_4_ NPs plus X‐ray irradiation (Figures 6f,g and S28, Supporting Information). Additionally, the infiltration of CD4^+^ and CD8^+^ T cells in tumor tissues was measured. After treatment of mice with Ov‐a/c‐CaWO_4_ NPs plus X‐ray, the percentages of CD4^+^ and CD8^+^ T cells in both primary and distant tumors were distinctly increased (Figures 6h,i and S29–S31, Supporting Information), which was consistent with immunofluorescence staining results (Figures S32 and S33, Supporting Information). Moreover, the secretion of immunostimulatory cytokines in the serum including IL‐6, IL‐12p70, TNF‐α, and interferon γ (IFN‐γ) was remarkably elevated in mice subjected to Ov‐a/c‐CaWO_4_ NPs injection and X‐ray irradiation (Figure 6j–m), further supporting the activation of antitumor immune responses.

CD8^+^ T cells, in addition to directly eliminating malignant cells, can provide long‐term protective immunity.^[^ ^20^ ^]^ To study the long‐term immune memory effects elicited by X‐ray‐activated Ov‐a/c‐CaWO_4_ NPs, the effector memory T cells (CD3^+^CD8^+^CD44^+^CD62L^−^) and central memory T cells (CD3^+^CD8^+^CD44^+^CD62L^+^) in spleens were analyzed on day 28 after treatment. As shown in Figure S34 (Supporting Information), the memory T cells in mice treated with Ov‐a/c‐CaWO_4_ NPs plus X‐ray irradiation were markedly increased compared with other groups. Following that, lung metastasis inhibition was examined by H&E staining after 28 days of treatment. Excitedly, no obvious metastatic nodules were observed in the lung tissues when mice were injected intravenously with Ov‐a/c‐CaWO_4_ NPs and then exposed to X‐ray irradiation at 0.5 Gy (Figure 6n). Taken together, the above results indicated that Ov‐a/c‐CaWO_4_ NPs‐mediated radiodynamic‐immunotherapy could effectively eliminate tumors, evoke systemic immune responses, and inhibit tumor metastasis.

## 3 Conclusion

In summary, amorphous/crystalline heterophase Ov‐a/c‐CaWO_4_ NPs with rich oxygen vacancies have been synthesized as an enhanced radiodynamic agent for tumor radiodynamic‐immunotherapy. Through simply changing the synthetic temperature from RT to 160 °C, a series of CaWO_4_ NPs with different phase composition and oxygen vacancy concentrations were obtained. Compared with amorphous and crystalline CaWO_4_ NPs, Ov‐a/c‐CaWO_4_ NPs prepared by one‐pot solvothermal method at 60 °C exhibited a superior radiodynamic effect because of their amorphous/crystalline heterophase interfaces and abundant oxygen vacancy defects, which facilitated charge carrier transfer and suppressed e^−^‐h^+^ recombination, respectively, enabling synergistically improved ROS generation from Ov‐a/c‐CaWO_4_ NPs under low‐dose X‐ray irradiation. Additionally, the radiodynamic production of ROS could not only directly cause oxidative damage of cancer cells but also elicit ICD to stimulate systemic antitumor immune responses, ultimately permitting the inhibition of both primary and distant tumors as well as cancer metastasis. This work presents the facile synthesis of oxygen vacancy‐rich amorphous/crystalline heterophase Ov‐a/c‐CaWO_4_ NPs for enhanced radiodynamic‐immunotherapy of cancer and provides a new strategy to synergistically promote the ROS‐generating ability of radiodynamic agents via the combination of phase and defect engineering.

## Conflict of Interest

The authors declare no conflict of interest.

## Supporting information



Supporting Information

## Data Availability

The data that support the findings of this study are available in the supplementary material of this article.
