# Supplementary material for: One‐Pot Synthesis of Oxygen Vacancy‐Rich Amorphous/Crystalline Heterophase CaWO4 Nanoparticles for Enhanced Radiodynamic‐Immunotherapy
Source: Adv Sci (Weinh). 2024 Dec 27;12(7):2409551. doi: 10.1002/advs.202409551 (PMC11831444; doi:10.1002/advs.202409551)
Supplement: Supplementary file 1 — Supporting Information [file ADVS-12-2409551-s001.docx]

Supporting Information

One-Pot Synthesis of Oxygen Vacancy-Rich Amorphous/Crystalline Heterophase CaWO_4_ Nanoparticles for Enhanced Radiodynamic-Immunotherapy

Shanshan Peng, Zhen Chen, Jun Wang, Meili Yu, Xuegang Niu, Tingting Cui, Rujiang Ao, Huilan Cai, Hongwei Huang, Lisen Lin*, Xiaoyuan Chen*, and Huanghao Yang*

Experimental Methods

*Chemicals*: Sodium tungstate dihydrate (NaWO_4_∙2H_2_O), calcium chloride (CaCl_2_), and ammonium hydroxide (NH_3_∙H_2_O, 25%-28%) were purchased from Sinopharm Chemical Reagents (Shanghai, China). Cyanine 5.5-polyethylene glycol-thiol (Cy5.5-PEG-SH) was bought from Xi’an Qiyue Biological Technology Co., Ltd (Xi’an, China). Polyethylene glycol 200 (PEG-200) and 2',7'-dichlorodihydrofluorescein (DCFH) were obtained from Yuanye Bio-Technology Co., Ltd. (Shanghai, China). Singlet oxygen sensor green (SOSG), aminophenyl fluorescein (APF), and dihydrorhodamine 123 (DHR 123) were purchased from Shanghai Maokang Bio-Technology Co., Ltd. (Shanghai, China). Fetal bovine serum (FBS) was bought from Thermo Fisher Scientific Inc. (Waltham, USA). Cell counting kit-8 (CCK-8) and calcein-AM/propidium iodide (PI) costaining kit were purchased from Dojindo Molecular Technologies, Inc. (Shanghai, China). Hoechst 33342, 2',7'-dichlorodihydrofluorescein diacetate (DCFH-DA), annexin V-FITC/PI apoptosis detection kit, acridine orange (AO), and adenosine triphosphate (ATP) assay kit were bought from Beyotime (Shanghai, China). The enzyme-linked immunosorbent assay (ELISA) kits for interleukin-6 (IL-6), interleukin-12p70 (IL-12p70), tumor necrosis factor-α (TNF-α) and interferon γ (IFN-γ) were acquired from Multi Sciences (Zhejiang, China). Anti-CD3ε-FITC (Catalog: 100305, Clone: 145-2C11), anti-CD3ε-PerCP-Cy5.5 (Catalog: 100327, Clone: 145-2C11), anti-CD4-PE (Catalog: 100408, Clone: GK1.5), anti-CD8a-APC (Catalog: 100712, Clone: 53-6.7), anti-CD80-PE (Catalog: 1004707, Clone: 16-10A1), anti-CD86-APC (Catalog: 105012, Clone: GL-1), anti-CD11c-FITC (Catalog: 117305, Clone: N418), anti-CD62L-FITC (Catalog: 1004405, Clone: MEL-14), and anti-CD44-PE (Catalog: 103007, Clone: IM7) antibodies were purchased from BioLegend, Inc. (California, USA). All chemicals were used without further purification, and deionized water was obtained from a Milli-Q water purification system.

*Instruments*: Transmission electron microscopy (TEM) images were recorded using HT7700 (Hitachi). Powder X-ray diffraction (XRD) patterns were obtained with a Bruker D8 Advance diffractometer (Germany). The scanning X-ray microprobe (PHI 5000 Verasa, ULAC-JY 2000-2, Horiba) was employed to perform X-ray photoelectron spectroscopy (XPS) analysis. The Fourier transform infrared (FT-IR) spectra were collected using a FT-IR spectrometer (Nicolet 5700). The electron paramagnetic resonance (EPR) measurements were carried out using a Bruker A300 spectrometer. Electrochemical impedance spectroscopy (EIS) spectra were obtained from electrochemical workstation (CHI660E). The fluorescence spectra were measured by Agilent spectrometer (G9800A). The fluorescence images of cells were acquired by confocal laser scanning microscope (Nikon C2). In vivo animal fluorescence images were taken using in vivo imaging system (IVIS, Perkin Elmer). The analysis of immune cells was performed by flow cytometry (CytoFLEX, Beckman).

*Synthesis of oxygen vacancy-rich amorphous/crystalline heterophase calcium tungstate nanoparticles (Ov-a/c-CaWO_4_ NPs)*: 1.0 mmol CaCl_2_ and 1.0 mmol NaWO_4_∙2H_2_O were dissolved in 1.0 mL and 2.3 mL of deionized water, respectively. Then, 16.7 mL of PEG-200 was added to a round bottom flask. Subsequently, the CaCl_2_ and NaWO_4_∙2H_2_O solutions were added in turn under vigorous stirring. After adjusting the pH to 9 with NH_3_∙H_2_O and stirring for 30 min, the mixture was transferred into a 25 mL Teflon reactor and kept at 60 ℃ for 14 h. The as-prepared Ov-a/c-CaWO_4_ NPs were washed and re-dispersed in deionized water. For comparison purpose, calcium tungstate nanoparticles (CaWO_4_ NPs) with different crystallinities including amorphous, amorphous/crystalline, and crystalline phases were also prepared at different solvothermal temperatures (room temperature (RT), 110 ℃, or 160 ℃) using the similar procedure.

*Synthesis of Cy5.5-labeled Ov-a/c-CaWO_4_ NPs*: 40 µg Cy5.5-PEG-SH was added into 10 mL of phosphate buffer saline (PBS) containing 10 mg Ov-a/c-CaWO_4_ NPs. After stirring for 24 h, the Cy5.5-labeled Ov-a/c-CaWO_4_ NPs were collected by centrifugation and washed three times with PBS.

*Detection of radiodynamic reactive oxygen species (ROS) generation from Ov-a/c-CaWO_4_ NPs*: The DCFH, which can be oxidized by ROS and then emits green fluorescence, was used as a fluorescent probe to detect the production of total ROS by Ov-a/c-CaWO_4_ NPs under low-dose X-ray irradiation. Briefly, an aqueous solution containing Ov-a/c-CaWO_4_ NPs (500 µg/mL) and DCFH (10 μM) was subjected to X-ray irradiation at a dose of 0.5 Gy, and then the fluorescence change was recorded.

In addition, the APF, DHR 123, and SOSG were employed to examine the generation of •OH, O_2_^•-^, and ^1^O_2_, respectively. The procedures were the same as above, except that DCFH was replaced by the corresponding specific dyes.

Intracellular radiodynamic ROS generation was evaluated using DCFH-DA probe. Briefly, 4T1 cells were incubated with Ov-a/c-CaWO_4_ NPs (100 μg/mL) for 12 h and then stained with DCFH-DA (10 μM) for 30 min. After X-ray irradiation at 0.5 Gy, Hoechst 33342 staining (10 μM, 30 min) was performed to indicate cell nuclei before observation with a confocal microscope.

*Lipid peroxidation (LPO) induced by X-ray-irradiated Ov-a/c-CaWO_4_ NPs*: The ability of Ov-a/c-CaWO_4_ NPs to induce LPO with the aid of X-ray irradiation was examined using C11-BODIPY^581/591^ ratiometric fluorescence probe. Briefly, 4T1 cells seeded in confocal dishes were incubated with Ov-a/c-CaWO_4_ NPs (100 μg/mL) for 12 h. After exposure to X-ray irradiation (0.5 Gy), the cells were stained with C11-BODIPY^581/591^ (10 μM) and Hoechst 33342 (10 μM) for 30 min, followed by confocal fluorescence imaging.

*Lysosomal damage initiated by X-ray-irradiated Ov-a/c-CaWO_4_ NPs*: 4T1 cells were plated in confocal dishes at 4×10^4^ cells/well. After 12 h of incubation with Ov-a/c-CaWO_4_ NPs (100 μg/mL), the cells were exposed to X-ray irradiation at 0.5 Gy and then stained with AO (5 μM) for 30 min prior to visualization by confocal microscopy.

*In vitro radiodynamic cytotoxicity of Ov-a/c-CaWO_4_ NPs against cancer cells*: 4T1 cells were seed into 96-well plates and cultured overnight. Subsequently, the cells were treated with different concentrations of Ov-a/c-CaWO_4_ NPs for 12 h and then subjected to X-ray irradiation at a dose of 0.5 Gy. After incubation for another 12 h, the culture medium was replaced with fresh medium containing 10 μL CCK-8 and the cells were further incubated for 1 h. Finally, the absorbance at 450 nm was measured to estimate cell viability.

The live/dead cell staining assay based on calcein-AM/PI was used to intuitively investigate the cancer cell killing effect of Ov-a/c-CaWO_4_ NPs. 4T1 cells seeded in 6-well plates were incubated with Ov-a/c-CaWO_4_ NPs (100 µg/mL) for 12 h, and then exposed to X-ray irradiation at 0.5 Gy. After being cultured for another 12 h, the cells were stained with calcein-AM (2 µg/mL) and PI (4.5 µg/mL) for 30 min and subsequently imaged with a fluorescence microscope.

Flow cytometry was employed for the analysis of cancer cell apoptosis triggered by the radiodynamic effect of Ov-a/c-CaWO_4_ NPs. 4T1 cells were seeded on 6-well plates and then incubated with Ov-a/c-CaWO_4_ NPs (100 µg/mL) for 12 h. After exposure to X-ray irradiation (0.5 Gy), the cells were cultured for another 12 h. Then, the cells were digested and collected, followed by staining with annexin V-FITC and PI according to the manufacturer’s instructions. Finally, the apoptotic cells were identified by flow cytometry.

*Immunofluorescence staining of high-mobility group box 1 (HMGB1) and calreticulin (CRT)*: Briefly, 4T1 cells seeded in confocal dishes were treated with Ov-a/c-CaWO_4_ NPs (100 µg/mL) for 12 h and then subjected to X-ray irradiation at a dose of 0.5 Gy. After incubation for another 12 h, the cells were fixed with 4% paraformaldehyde for 10 min at room temperature and then permeabilized with 1% Triton X-100 for 10 min. After being washed three times with PBS, the cells were blocked with 10% goat serum for 30 min. Following that, the cells were incubated with HMGB1 rabbit monoclonal antibody at 37 ℃ for 1 h, and then washed with Tris-buffered saline Tween-20 (TBST) solution for three times. Subsequently, Alexa Fluor 561-conjugated goat anti-rabbit IgG and Hoechst 33342 were added to incubate for 30 min, and the fluorescence images were recorded. Similarly, the immunofluorescence staining of CRT was carried out by the same procedure except that HMGB1 rabbit monoclonal antibody was replaced by CRT rabbit monoclonal antibody.

*Detection of ATP release induced by X-ray-irradiated Ov-a/c-CaWO_4_ NPs*: 4T1 cells cultured in 6-well plates were treated with Ov-a/c-CaWO_4_ NPs (100 µg/mL) for 12 h. After X-ray irradiation (0.5 Gy), the cells were cultured for another 12 h. Finally, the cell culture medium was collected and the released ATP was detected by ATP assay kit.

*In vitro maturation of dendritic cells (DCs)*: 4T1 cells seeded in transwell chambers were treated with Ov-a/c-CaWO_4_ NPs (100 µg/mL) for 12 h, followed by X-ray irradiation (0.5 Gy). Then, the pre-treated 4T1cells were co-cultured with immature DCs in the transwell system for another 12 h. Subsequently, DCs were collected for flow cytometry analysis after staining with anti-CD80-PE, anti-CD86-APC, and anti-CD11c-FITC antibodies. Also, the culture supernatant of DCs was collected to measure the secretion of pro-inflammatory cytokines including IL-6, IL-12p70, and TNF-α by corresponding ELISA kits.

*In vivo radiodynamic-immunotherapy of cancer with Ov-a/c-CaWO_4_ NPs*: Animal experiments were performed according to the protocols approved by the Institutional Animal Care and Use Committee of Fuzhou University and Fujian Medical University (IACUC FJMU 2022-0608). 4T1 cells (1×10^6^ in PBS) were subcutaneously injected into the right lower limb of female BALB/c mice (6-8 weeks) to establish the tumor xenograft model. When tumor volume reached approximately 80 mm^3^, the mice were randomly divided into four groups (6 mice per group) treated with PBS, X-ray irradiation, Ov-a/c-CaWO_4_ NPs, or Ov-a/c-CaWO_4_ NPs plus X-ray irradiation. The X-ray irradiation (0.5 Gy) was performed after 12 h of intravenous injection with Ov-a/c-CaWO_4_ NPs (25 mg/kg). The body weight and tumor volume were recorded every two days. Tumor volume = (tumor length) × (tumor width)^2^/2. All major organs and tumors were collected and further analyzed after 14 days of treatment.

For bilateral tumor model, female BALB/c mice (6-8 weeks) were subcutaneously injected with 1×10^6^ 4T1 cells into the right flank (primary tumor), followed by the subcutaneous injection of 5×10^5^ 4T1 cells into the left flank (distant tumor) 6 days later. Then, the mice were randomly divided into four groups (6 mice per group) treated with PBS, X-ray irradiation, Ov-a/c-CaWO_4_ NPs, or Ov-a/c-CaWO_4_ NPs plus X-ray irradiation. After 12 h of intravenous injection with Ov-a/c-CaWO_4_ NPs (25 mg/kg), only the primary tumors were exposed to X-ray irradiation at a dose of 0.5 Gy. The tumor volume and body weight were monitored every two days for 14 days.

To assess the maturation status of DCs in tumor-draining lymph nodes, the mice were sacrificed at day 14 after treatment and tumor-draining lymph nodes were harvested for analysis. The tumor-draining lymph nodes were ground in PBS followed by filtration to obtain single-cell suspensions. The single-cell suspensions were stained with anti-CD80-PE, anti-CD86-APC, and anti-CD11c-FITC antibodies for flow cytometry analysis.

To analyze CD4^+^ and CD8^+^ T cells in tumor tissues, the mice were sacrificed on day 14 post-treatment. The bilateral tumors were collected and triturated into single-cell suspensions. The single-cell suspensions of primary and distant tumors were stained with anti-CD3ε-FITC plus anti-CD8a-APC antibodies or anti-CD3ε-FITC plus anti-CD4-PE antibodies for flow cytometry analysis.

To evaluate splenic activated CD4^+^ and CD8^+^ T cells, the mice were euthanized after 14 days of treatment and the spleens harvested from mice were ground in PBS. Subsequently, the spleen mixture was centrifuged and then resuspended in red blood cell lysis solution for 5 min. After washing with PBS for three times and filtration through a 70 μm cell strainer, the single-cell suspension was stained with anti-CD3ε-FITC plus anti-CD8a-APC antibodies or anti-CD3ε-FITC plus anti-CD4-PE antibodies prior to flow cytometry analysis.

To detect the secretion of immunostimulatory cytokines, the serum of mice was obtained at day 14. The levels of IL-6, TNF-α, IL-12p70, and IFN-γ were determined by corresponding ELISA kits according to the manufacturer’s protocols.

To analyze memory T cells, spleens were collected on day 28 after treatment and mechanically homogenized for single-cell suspension. After staining with anti-CD3ε-PerCP-Cy5.5, anti-CD8a-APC, anti-CD44-PE, and anti-CD62L-FITC antibodies, the single-cell suspension was subjected to flow cytometry analysis.

*Statistical analysis*: All quantitative data are displayed as mean ± standard deviation (SD). Origin 2019b software was used for statistical analysis. Statistical significance was calculated using the Student’s two-sided t-test. *P < 0.05, **P < 0.01, ***P < 0.001.


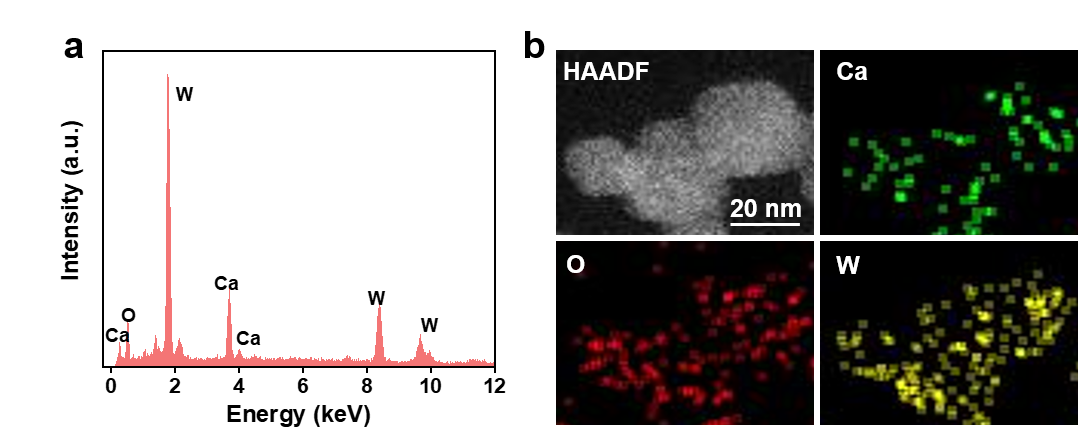


**Figure S1.** a) Energy-dispersive X-ray spectroscopy (EDS) spectrum and b) EDS mapping of Ov-a/c-CaWO_4_ NPs.


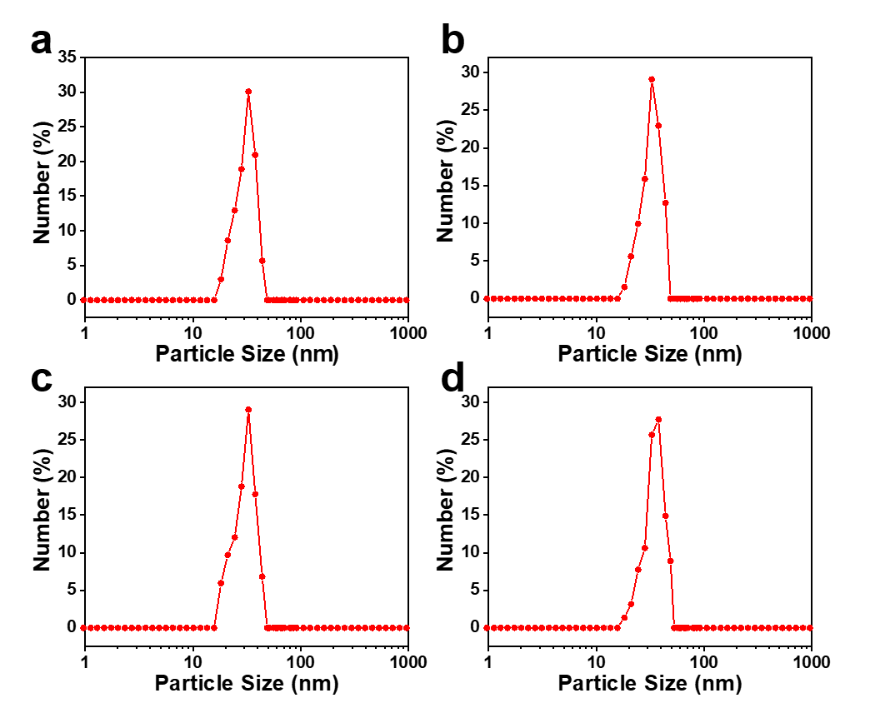


**Figure S2.** Dynamic light scattering (DLS) size distribution of CaWO_4_ NPs prepared at a) RT, b) 60 °C, c) 110 °C, or d) 160 °C.


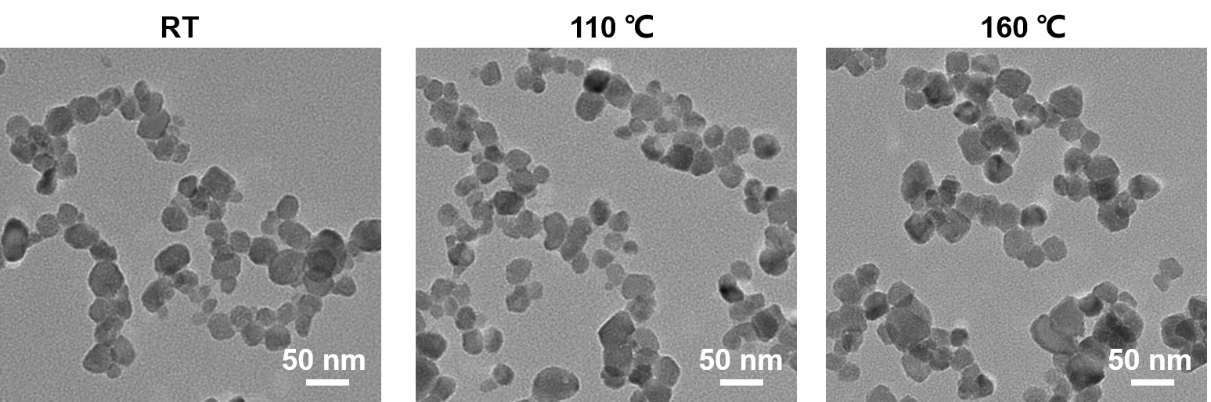


**Figure S3.** TEM images of CaWO_4_ NPs prepared at different temperatures including RT, 110 °C, or 160 °C.


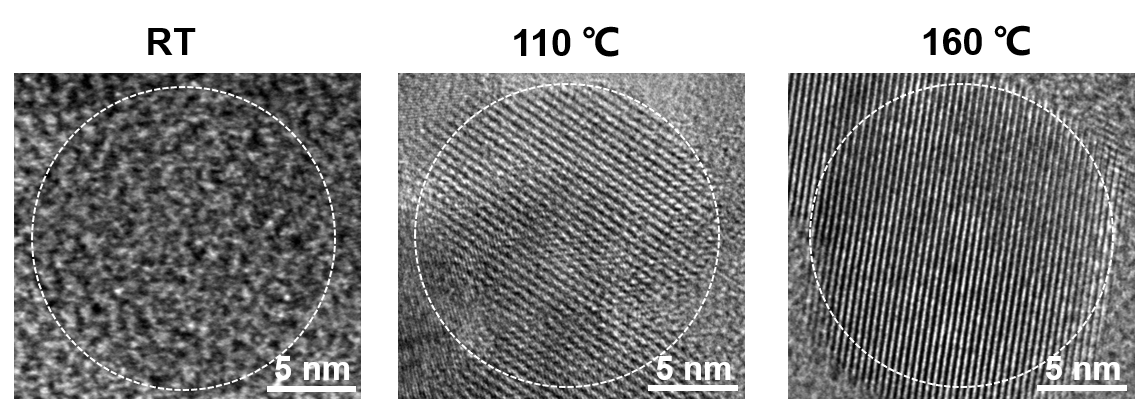


**Figure S4.** High-resolution TEM images of CaWO_4_ NPs synthesized at various temperatures including RT, 110 °C, or 160 °C.


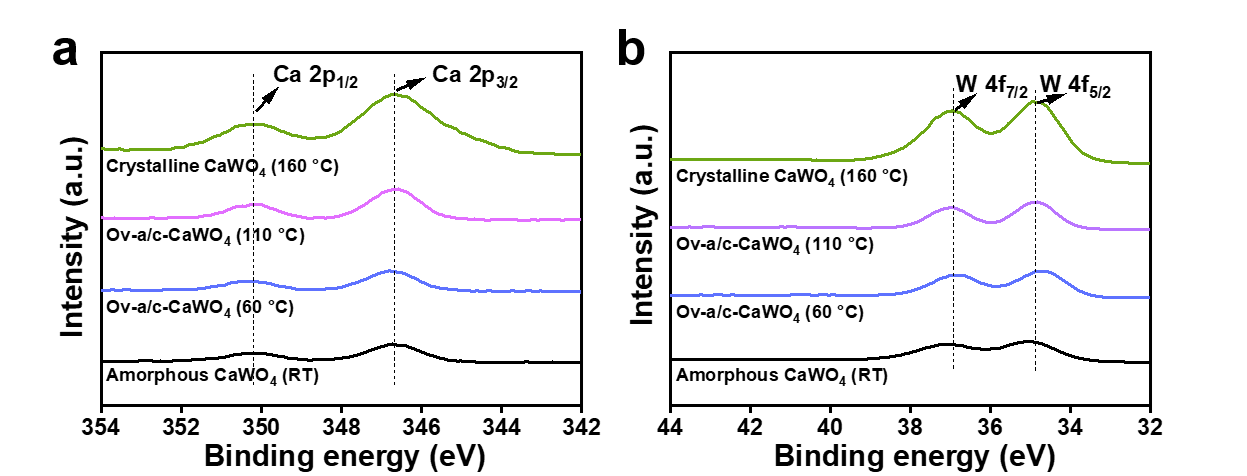


**Figure S5.** a) Ca 2p and b) W 4f XPS spectra of CaWO_4_ NPs prepared at different temperatures including RT, 60 °C, 110 °C, or 160 °C.


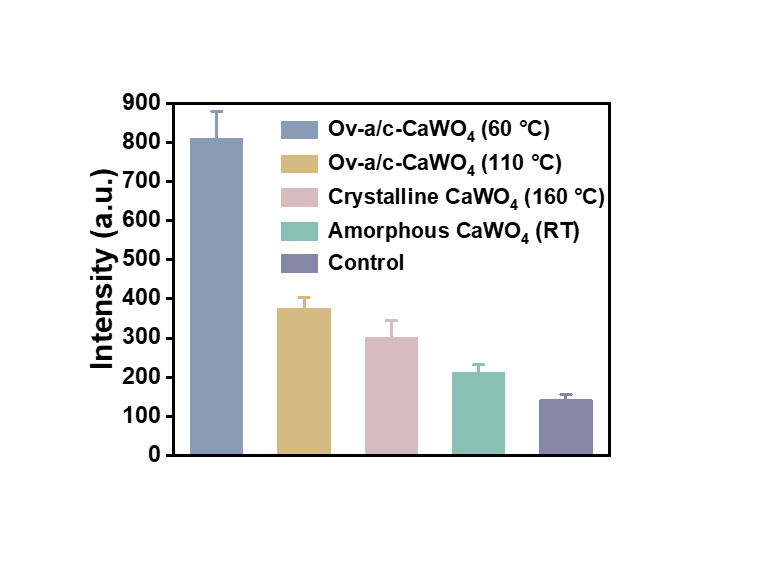


**Figure S6.** Fluorescence intensity of DCFH mixed with various CaWO_4_ NPs synthesized at different temperatures (including RT, 60 °C, 110 °C, or 160 °C) after X-ray irradiation (0.5 Gy). n = 3. Data are presented as mean ± SD.


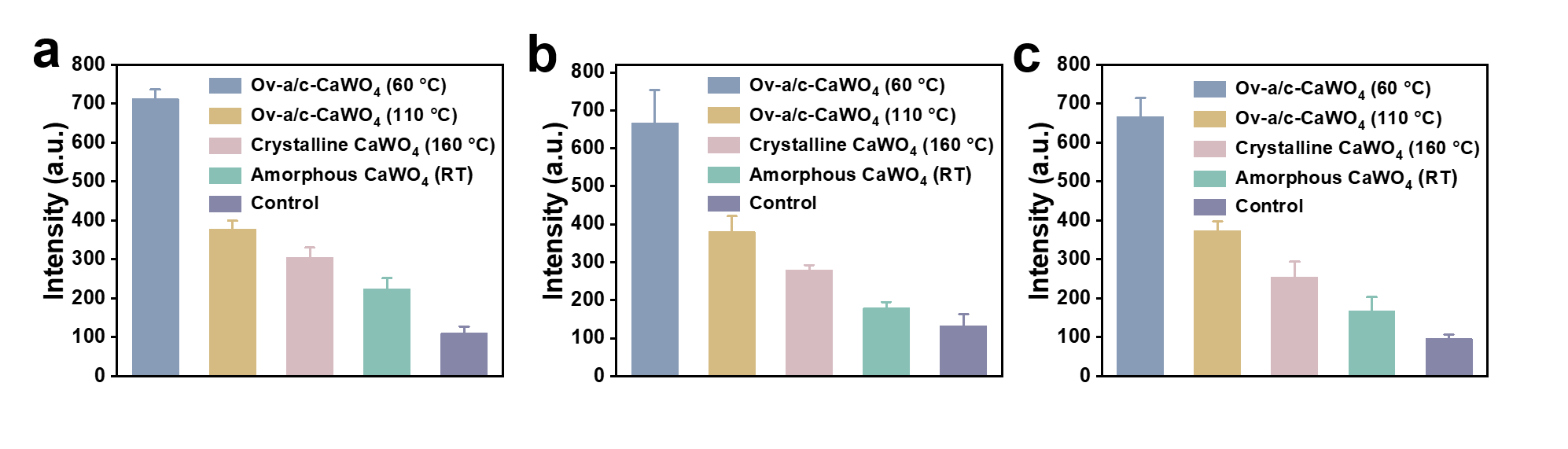


**Figure S7.** The generation of a) •OH, b) O_2_^•-^, and c) ^1^O_2_ from diverse CaWO_4_ NPs under X-ray irradiation at 0.5 Gy, measured by APF, DHR 123, and SOSG, respectively. n = 3. Data are presented as mean ± SD.


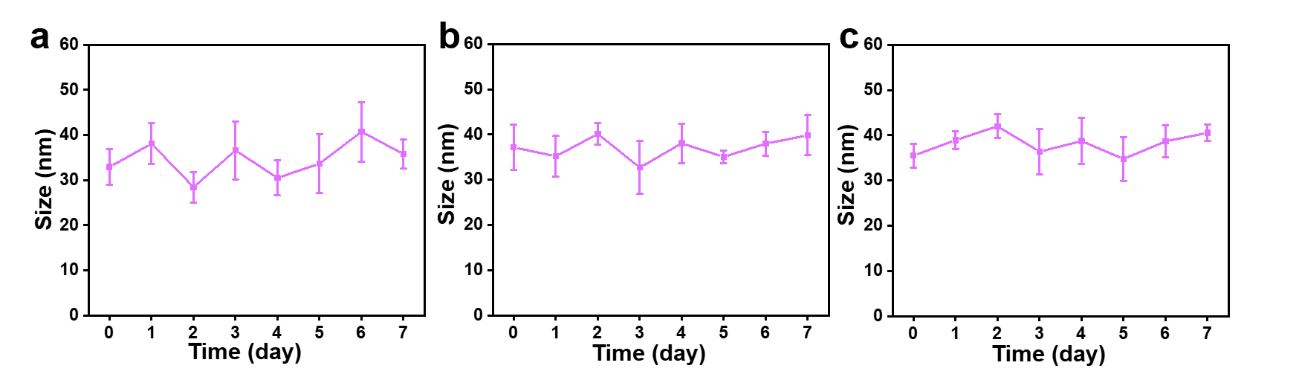


**Figure S8.** DLS sizes of Ov-a/c-CaWO_4_ NPs dispersed in a) deionized water, b) PBS, and c) cell culture medium plus 10% FBS at different time points. n = 3. Data are presented as mean ± SD.


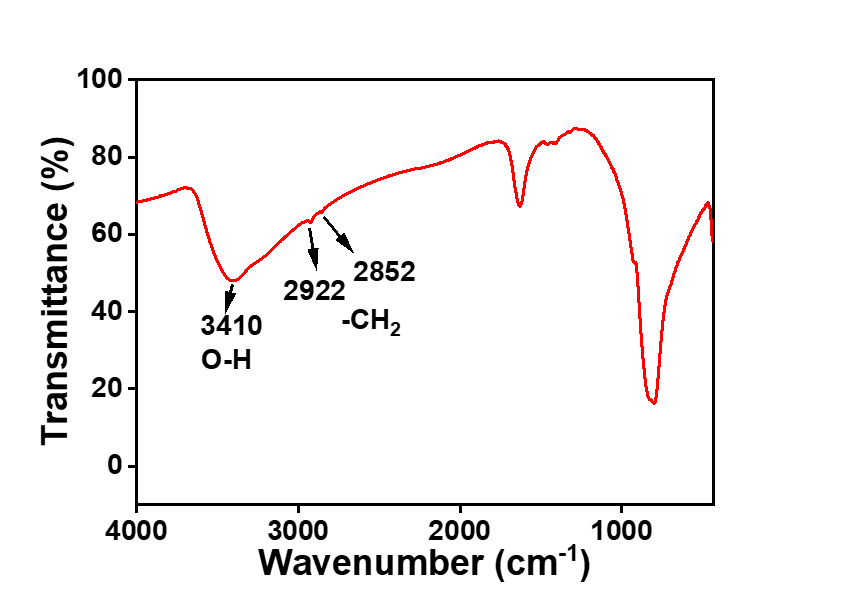


**Figure S9.** FT-IR spectrum of PEG-200-coated Ov-a/c-CaWO_4_ NPs.


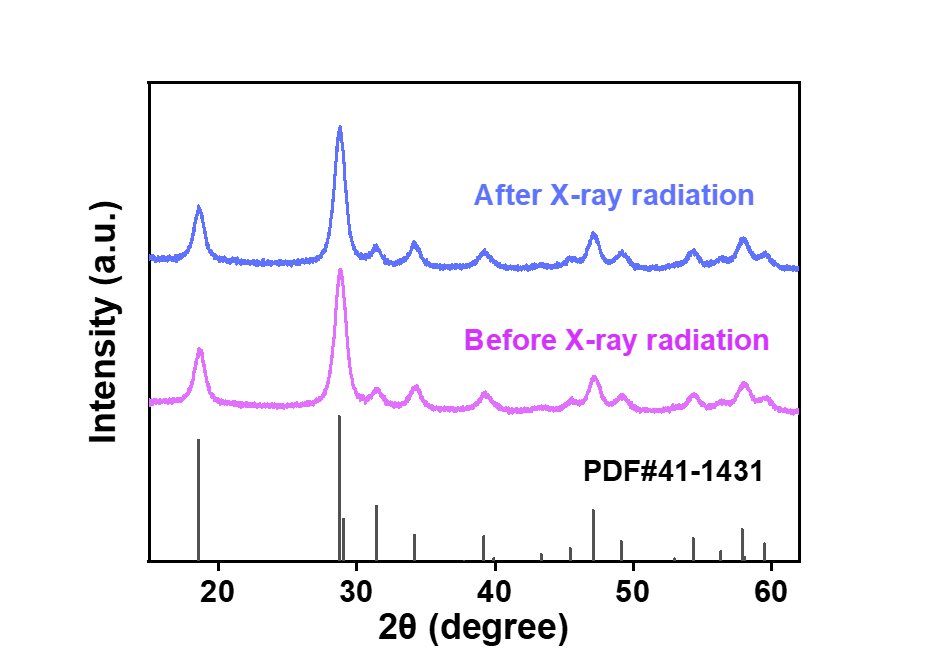


**Figure S10.** XRD patterns of Ov-a/c-CaWO_4_ NPs before and after X-ray irradiation.


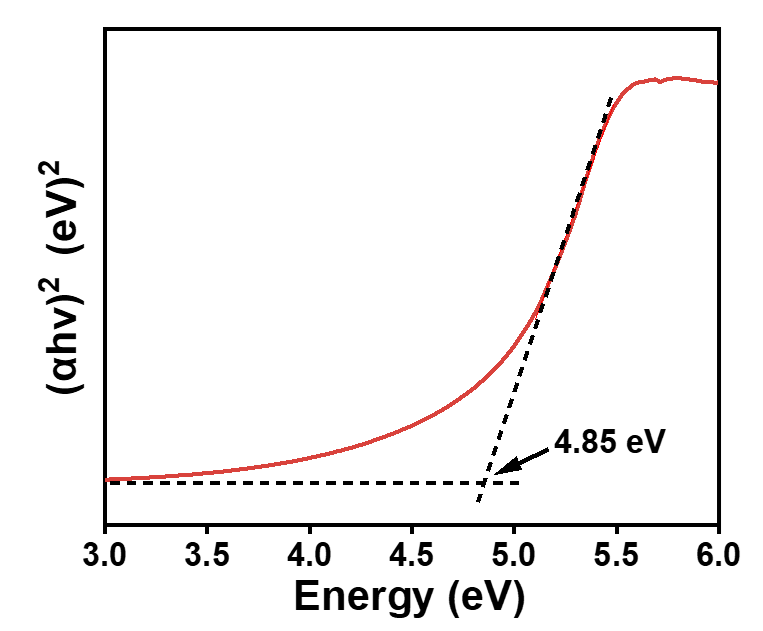


**Figure S11.** UV−Vis diffuse reflectance spectrum (DRS) of Ov-a/c-CaWO_4_ NPs.


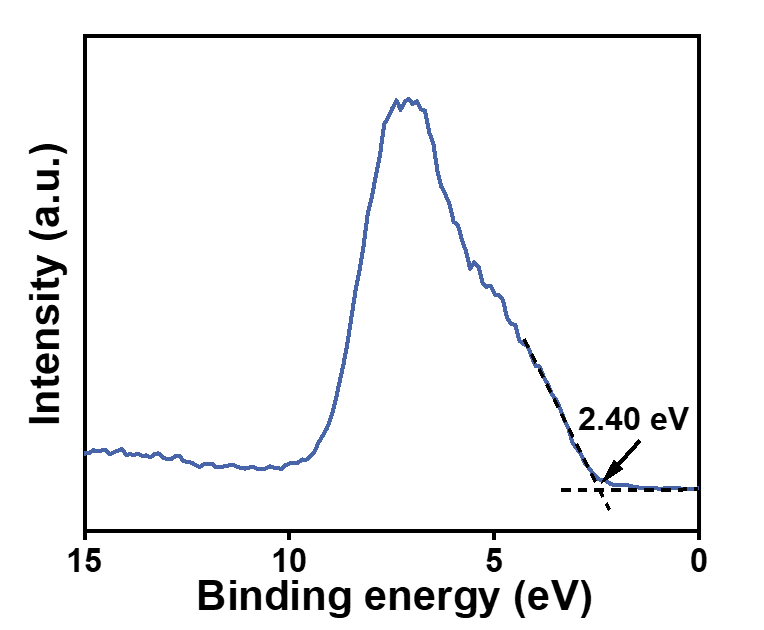


**Figure S12.** Valence band XPS (VB-XPS) spectrum of Ov-a/c-CaWO_4_ NPs.


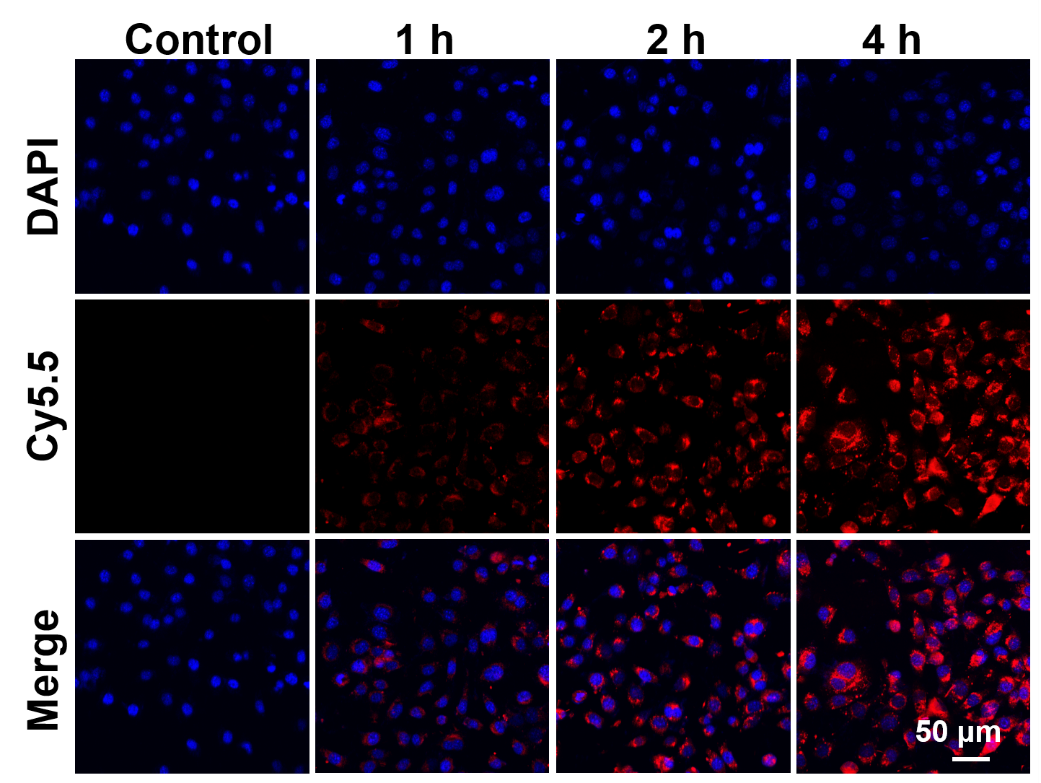


**Figure S13.** Fluorescence images of 4T1 cells after incubation with Cy5.5-labeled Ov-a/c-CaWO_4_ NPs at various time points.


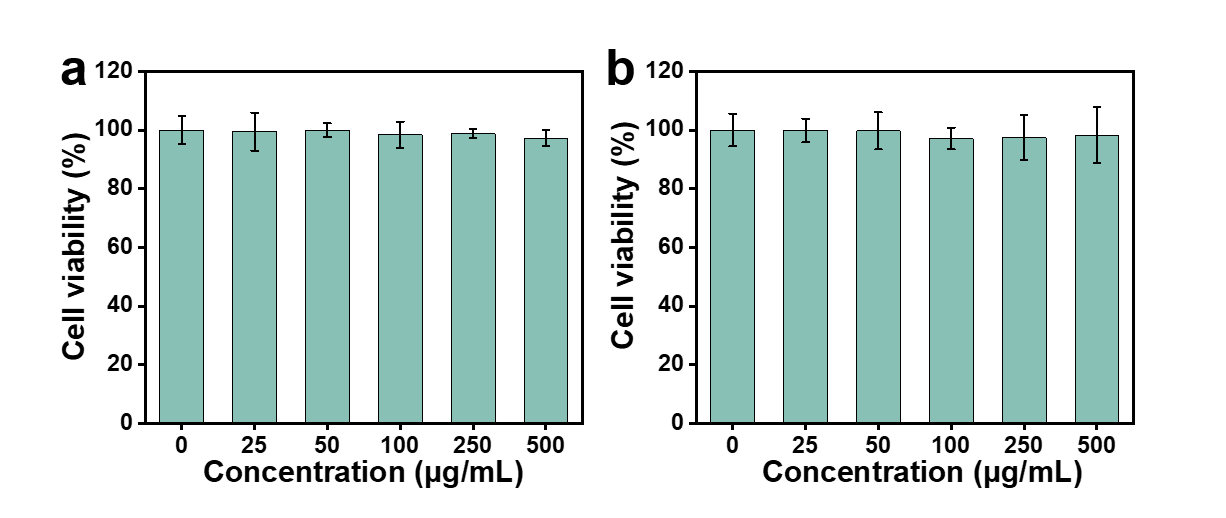


**Figure S14.** Cell viability of a) 4T1 and b) L02 cells exposed to different concentrations of Ov-a/c-CaWO_4_ NPs for 24 h. n = 3. Data are presented as mean ± SD.

**
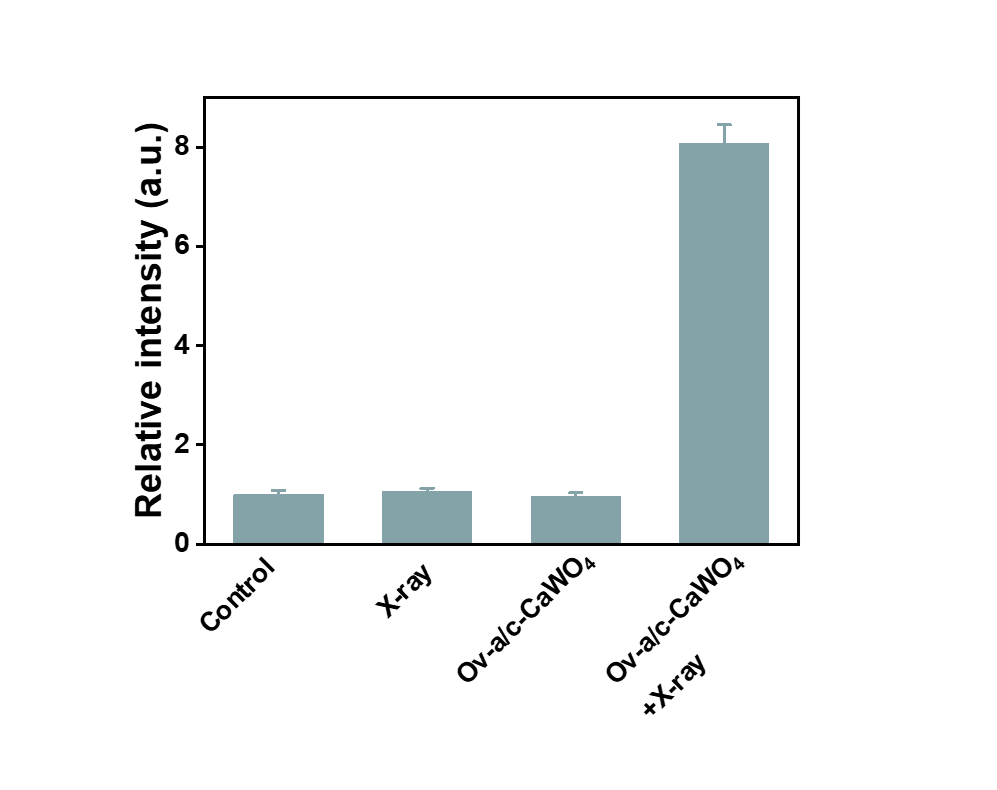
**

**Figure S15.** Relative fluorescence intensity of 4T1 cells stained with DCFH-DA after exposure to X-ray irradiation, Ov-a/c-CaWO_4_ NPs, or Ov-a/c-CaWO_4_ NPs plus X-ray irradiation (0.5 Gy). n = 3. Data are presented as mean ± SD.


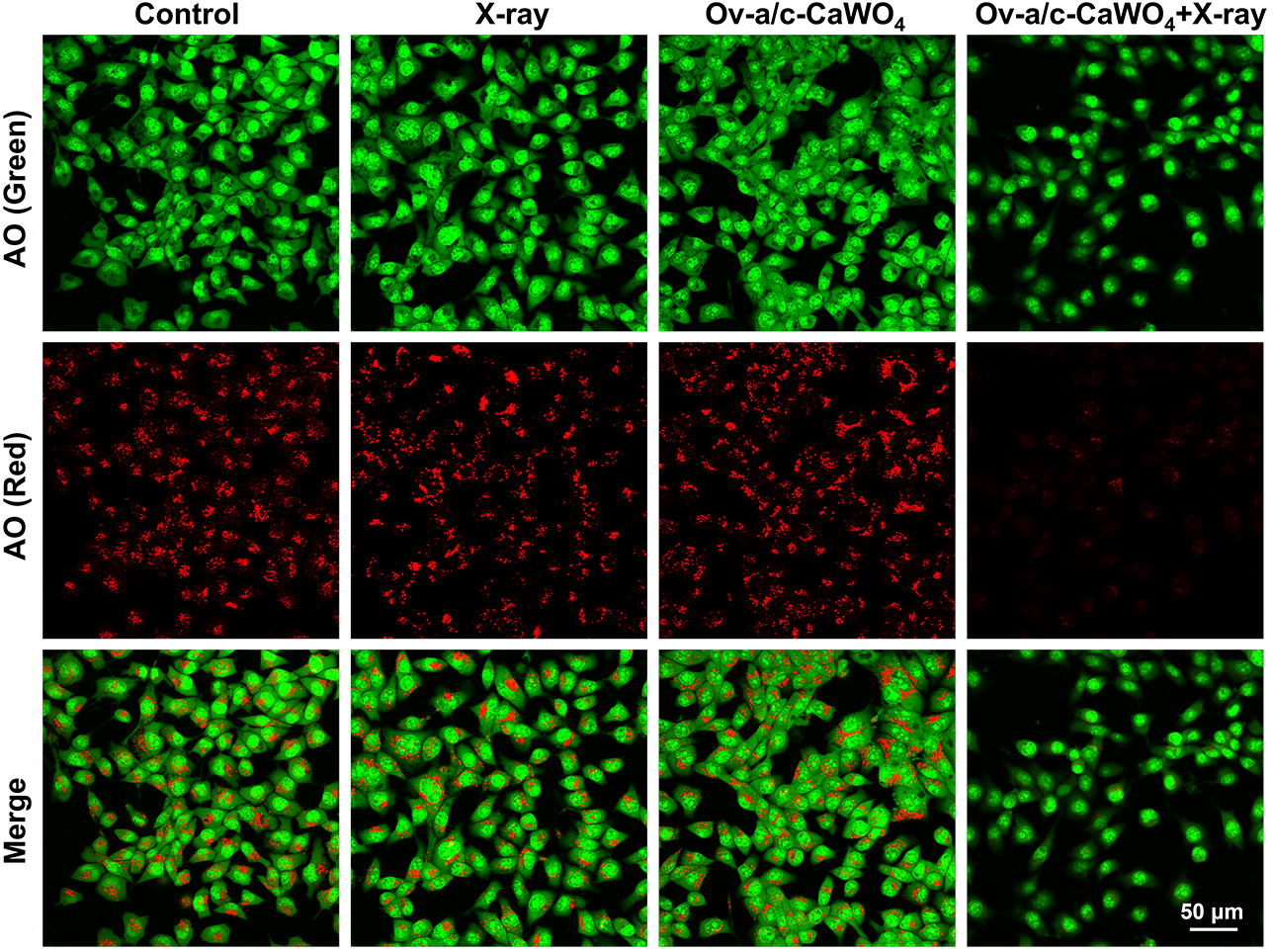


**Figure S16.** Fluorescence images of 4T1 cells stained with AO after exposure to X-ray irradiation, Ov-a/c-CaWO_4_ NPs, or Ov-a/c-CaWO_4_ NPs plus X-ray irradiation (0.5 Gy).


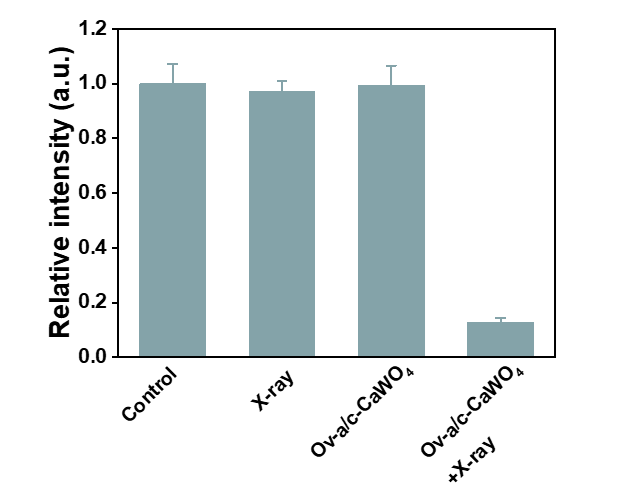


**Figure S17.** Relative immunofluorescence intensity of HMGB1 in 4T1 cells treated with X-ray irradiation, Ov-a/c-CaWO_4_ NPs, or Ov-a/c-CaWO_4_ NPs plus X-ray irradiation (0.5 Gy). n = 3. Data are presented as mean ± SD.


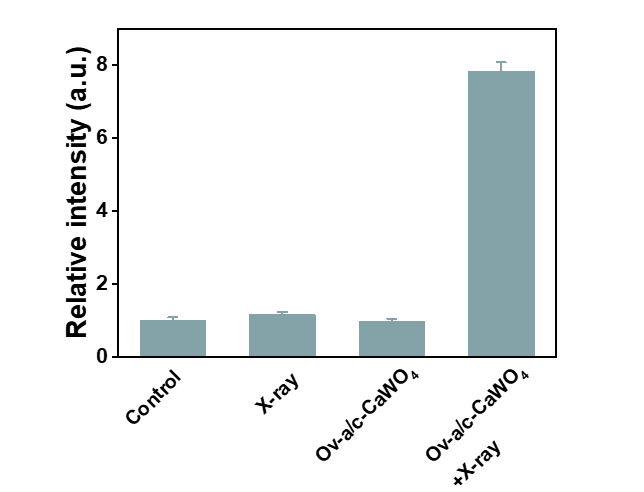


**Figure S18.** Relative immunofluorescence intensity of CRT exposure in 4T1 cells treated with X-ray irradiation, Ov-a/c-CaWO_4_ NPs, or Ov-a/c-CaWO_4_ NPs plus X-ray irradiation (0.5 Gy). n = 3. Data are presented as mean ± SD.


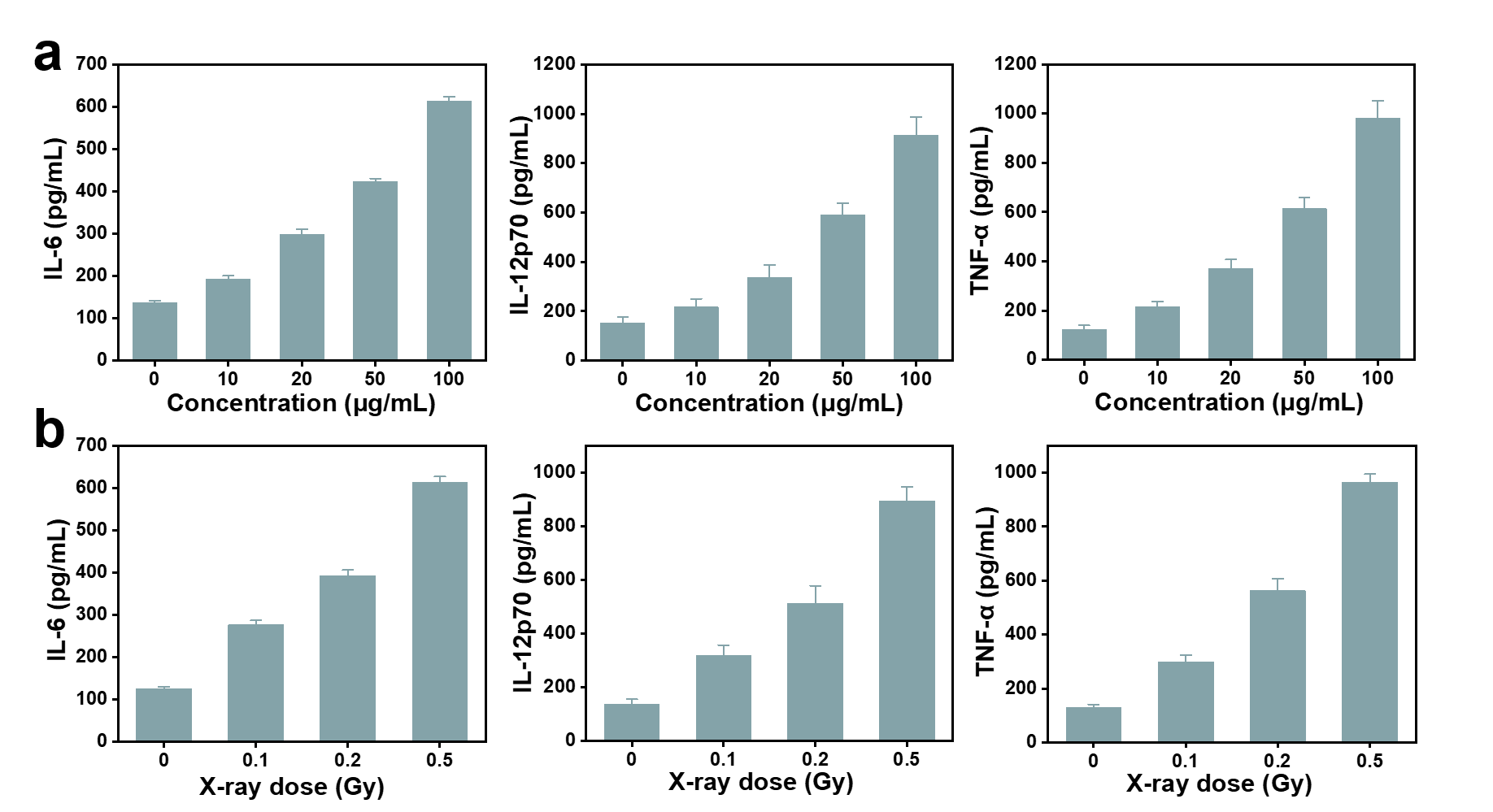


**Figure S19.** The secretion of IL-6, IL-12p70, and TNF-α in cell culture medium from various groups of DCs after exposure to 4T1 cells pre-treated with a) different concentrations of Ov-a/c-CaWO_4_ NPs plus X-ray irradiation (0.5 Gy) or b) 100 µg/mL Ov-a/c-CaWO_4_ NPs plus different doses of X-ray irradiation. n = 3. Data are presented as mean ± SD.


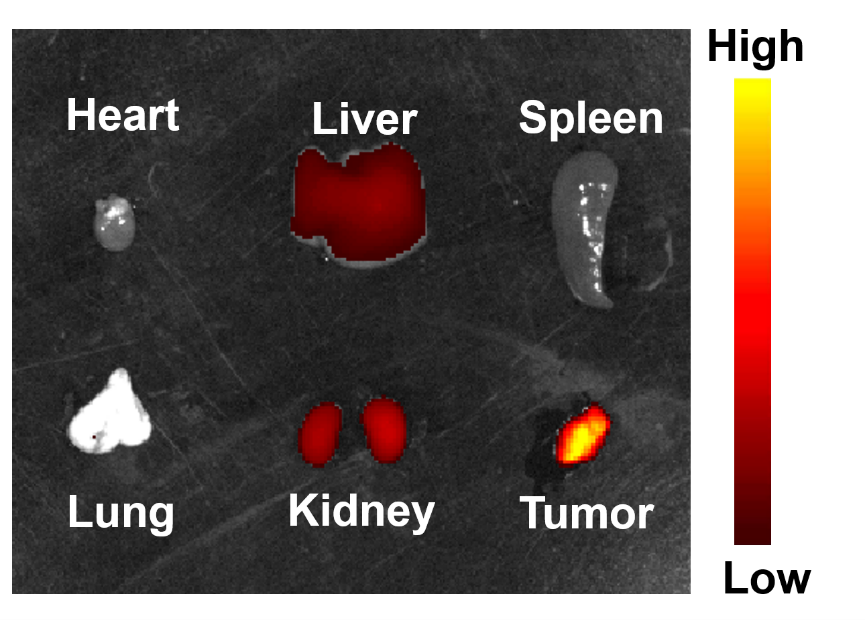


**Figure S20.** Fluorescence image of major organs and tumor at 12 h post-injection of Cy5.5-labeled Ov-a/c-CaWO_4_ NPs.


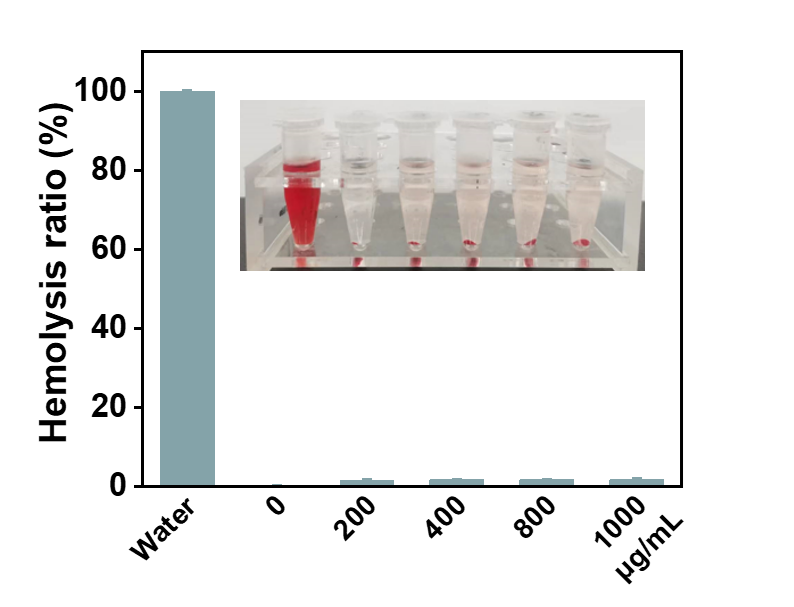


**Figure S21.** Hemolysis in response to different concentrations of Ov-a/c-CaWO_4_ NPs (Inset: photograph of centrifuge tubes containing the supernatant from erythrocytes exposed to deionized water or different concentrations of Ov-a/c-CaWO_4_ NPs in PBS). n = 3. Data are presented as mean ± SD.


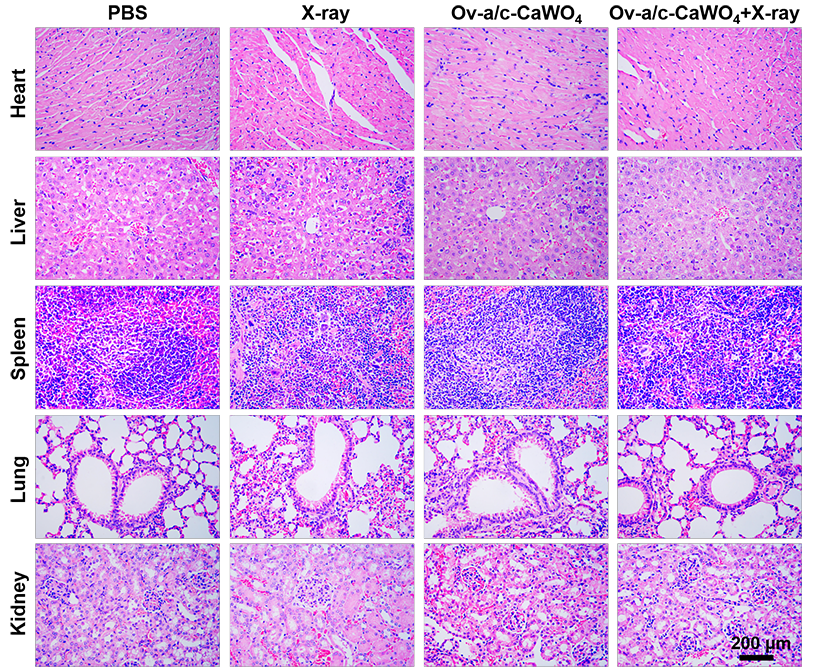


**Figure S22.** Hematoxylin and eosin (H&E) staining of major organs harvested from mice in various groups after 14 days of treatment.


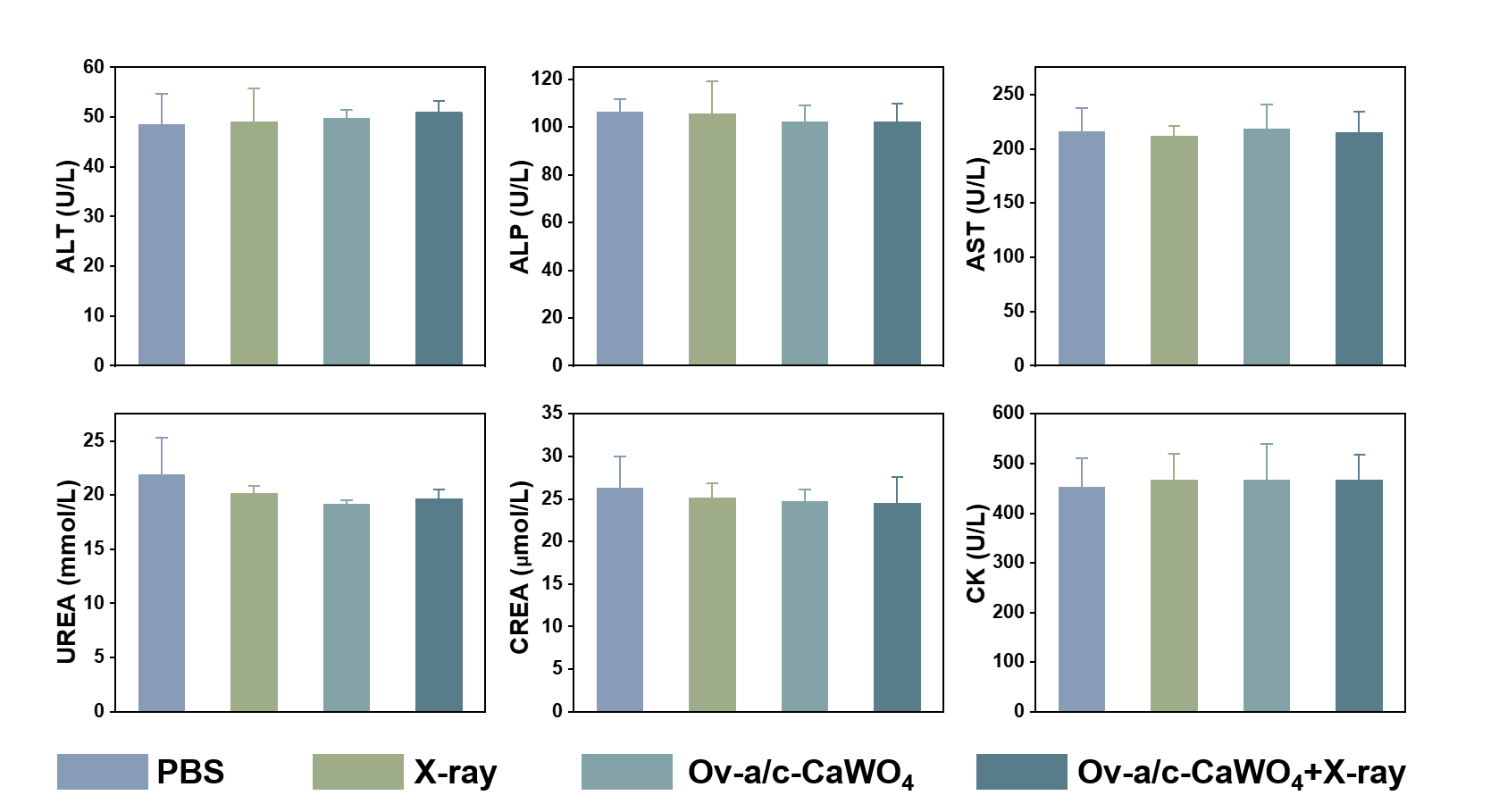


**Figure S23.** Blood biochemistry analysis of mice in different groups on day 14 post-treatment. ALT, alanine transferase; ALP, alkaline phosphatase; AST, aspartate transferase; UREA, urea; CREA, creatinine; CK, creatine kinase. n = 3. Data are presented as mean ± SD.


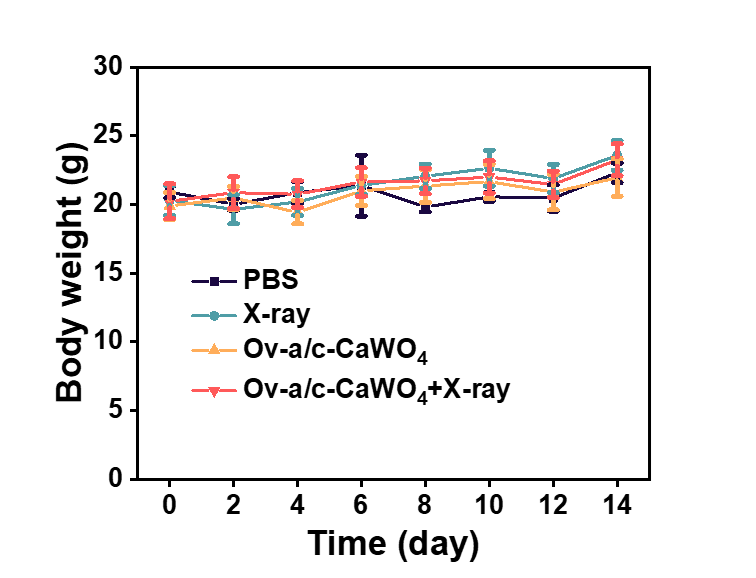


**Figure S24.** Body weight curves of bilateral 4T1 tumor-bearing mice during the treatment period. n = 6. Data are presented as mean ± SD.


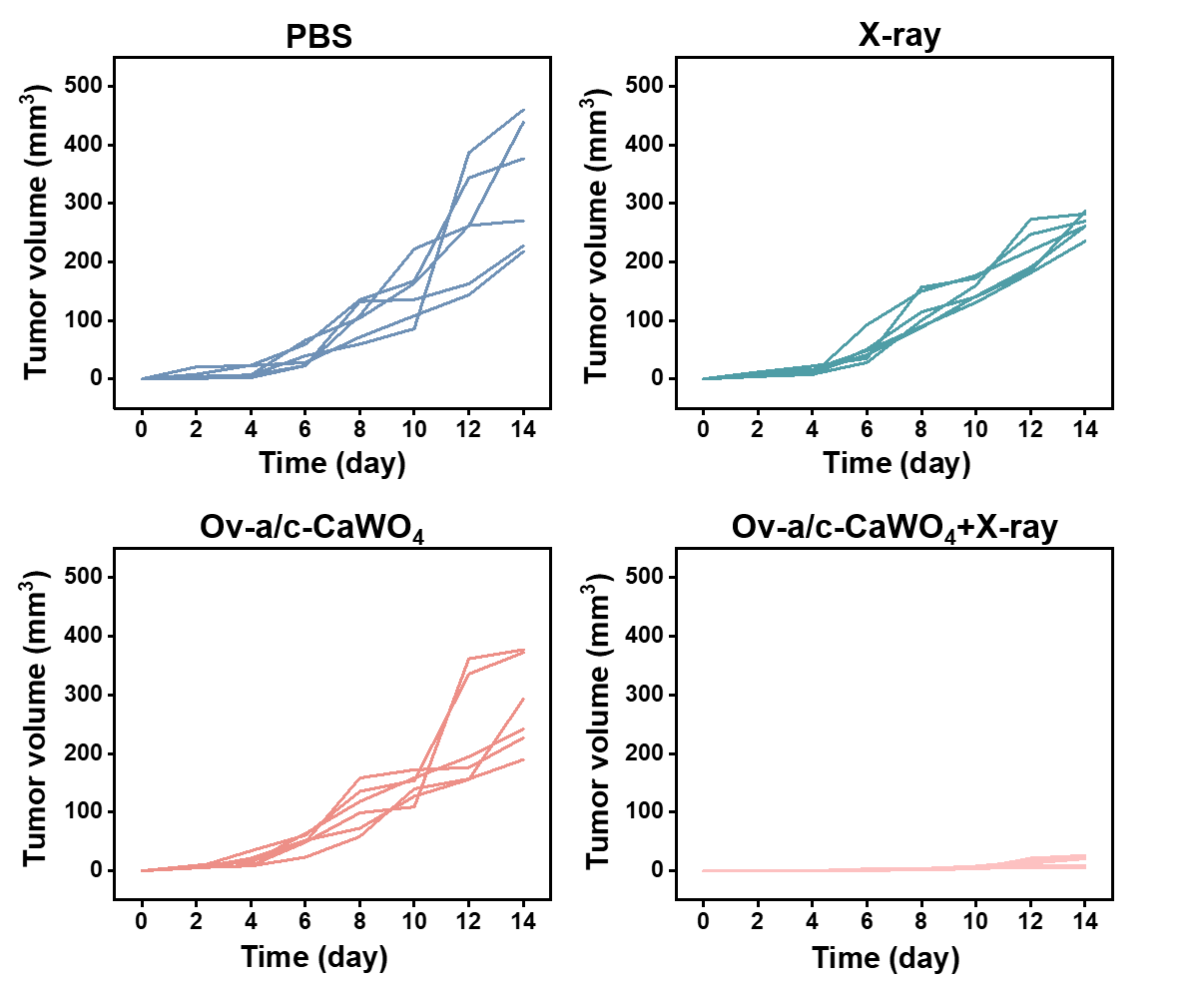


**Figure S25.** Individual growth curves of distant tumors in bilateral 4T1 tumor-bearing mice after treatment with PBS, X-ray irradiation, Ov-a/c-CaWO_4_ NPs, or Ov-a/c-CaWO_4_ NPs plus X-ray irradiation.


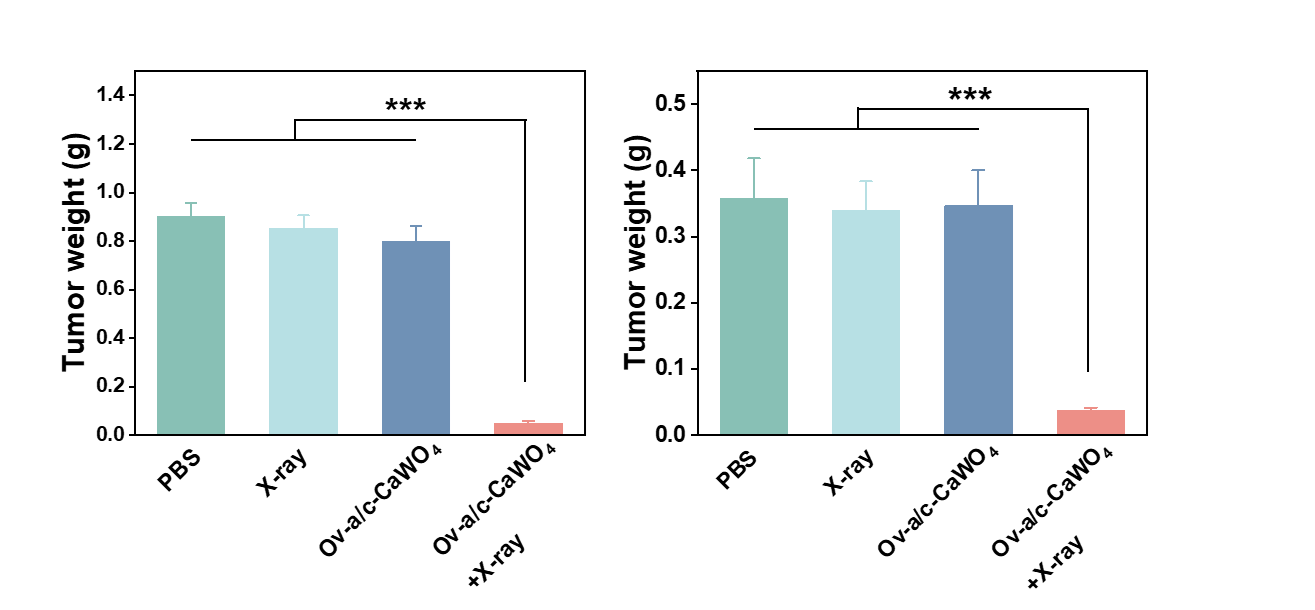


**Figure S26.** Tumor weight of a) primary and b) distant tumors in bilateral 4T1 tumor-bearing mice after treatment with PBS, X-ray irradiation, Ov-a/c-CaWO_4_ NPs, or Ov-a/c-CaWO_4_ NPs plus X-ray irradiation. n = 6. Data are presented as mean ± SD. ***P < 0.001.


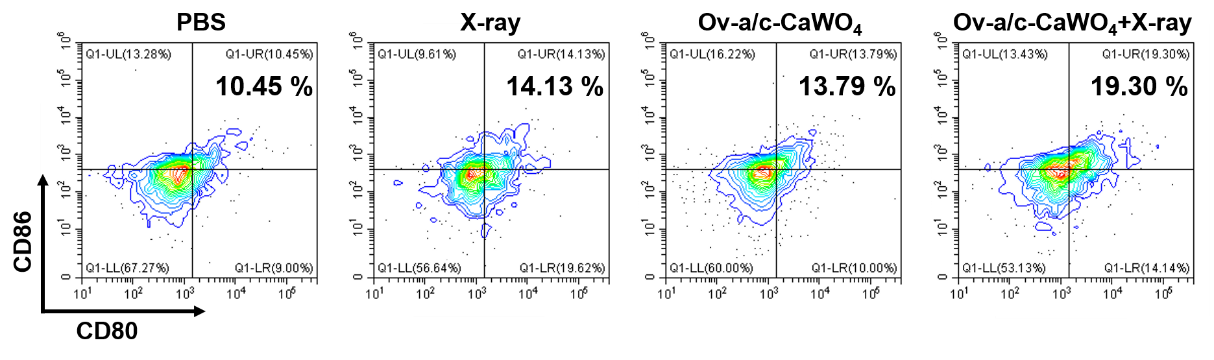


**Figure S27.** Flow cytometry analysis of CD80^+^CD86^+^ DCs in tumor-draining lymph nodes of mice after various treatments (gated on CD11c^+^ cells).


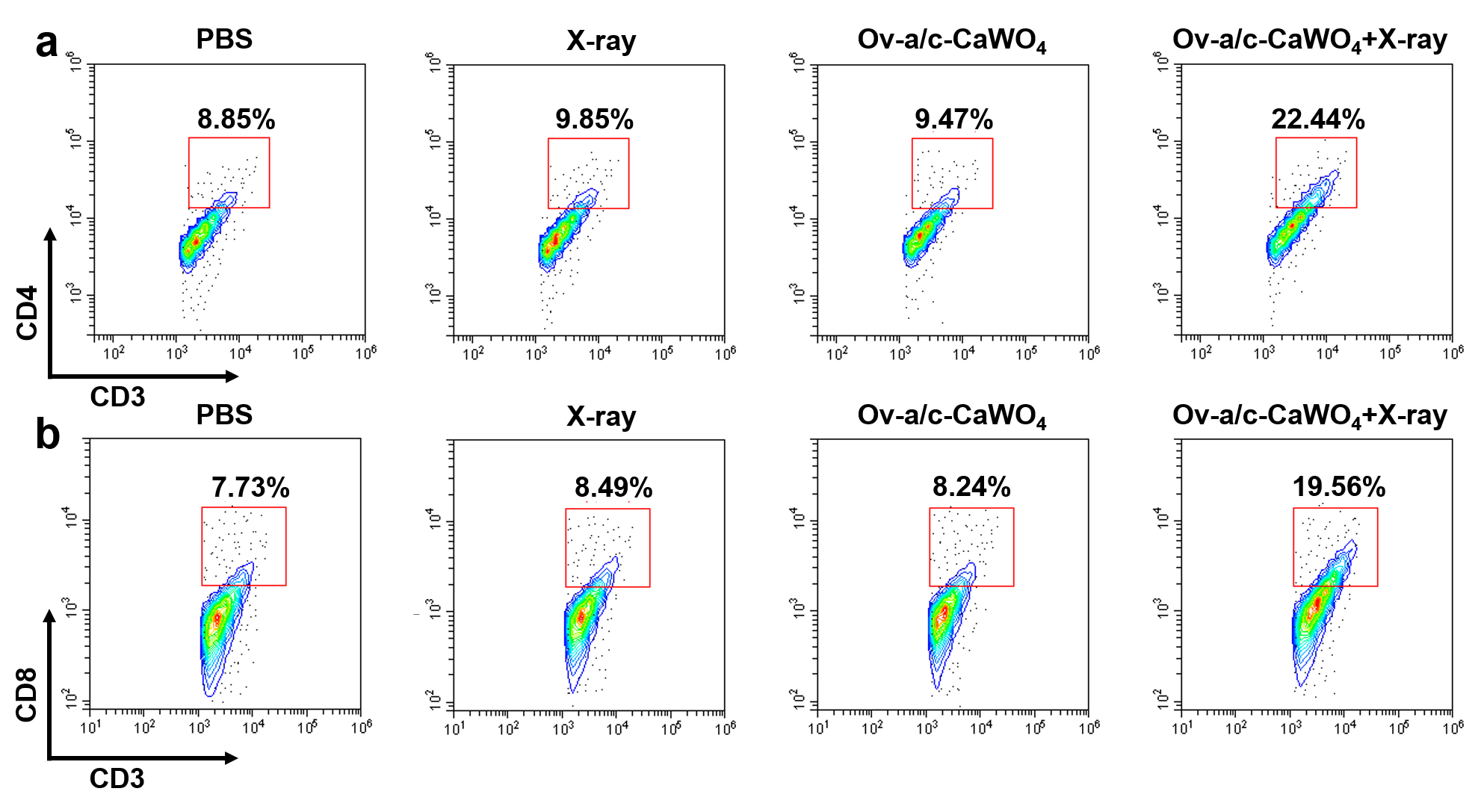


**Figure S28.** Flow cytometry analysis of a) CD4^+^ T cells (CD3^+^CD4^+^) and b) CD8^+^ T cells (CD3^+^CD8^+^) in spleens of different groups of mice (gated on CD3^+^ cells).


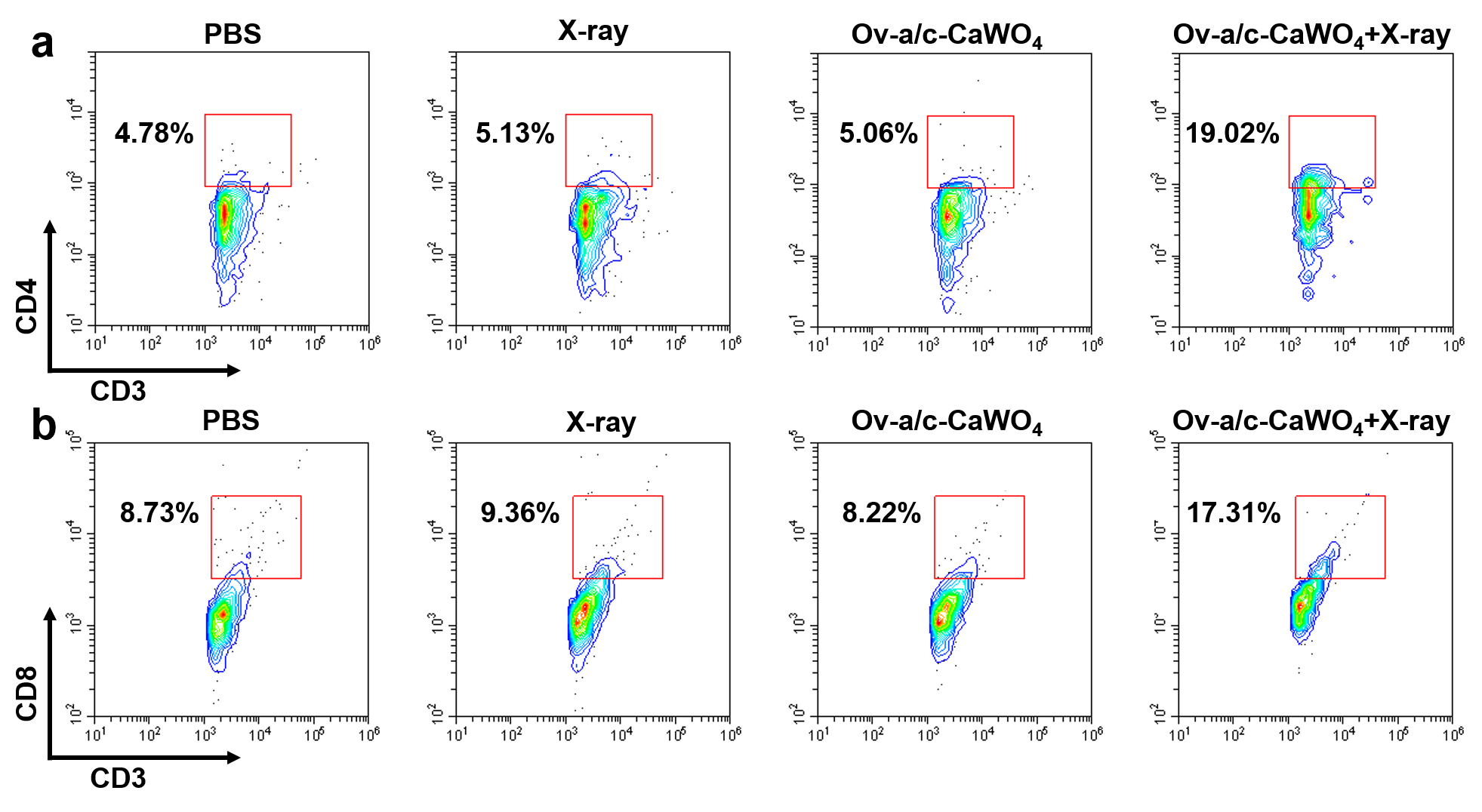


**Figure S29.** Flow cytometry analysis of a) CD4^+^ T cells (CD3^+^CD4^+^) and b) CD8^+^ T cells (CD3^+^CD8^+^) in primary tumors of bilateral 4T1 tumor-bearing mice after treatment with PBS, X-ray irradiation, Ov-a/c-CaWO_4_ NPs, or Ov-a/c-CaWO_4_ NPs plus X-ray irradiation (gated on CD3^+^ cells).


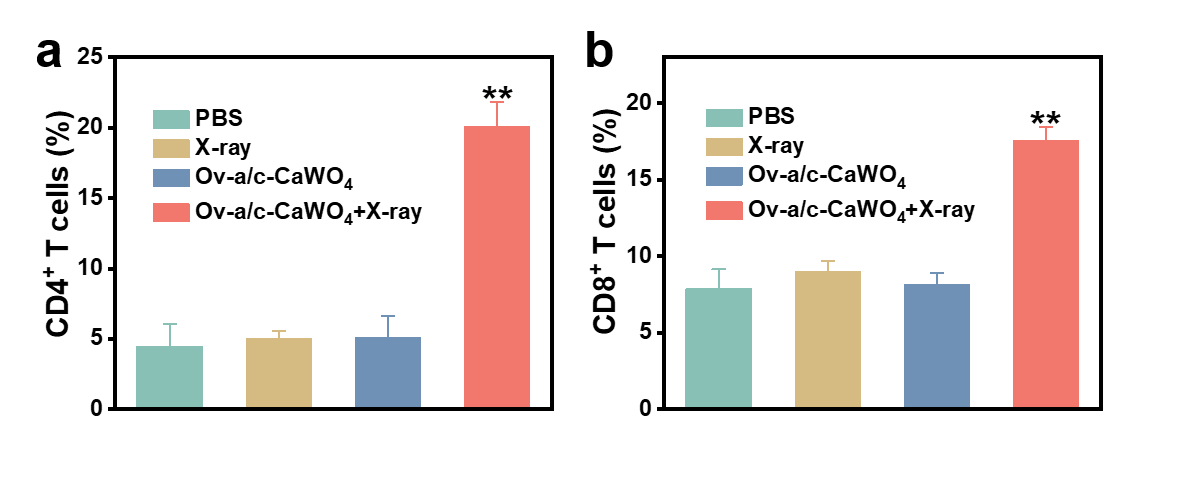


**Figure S30.** Quantitative analysis by flow cytometry of a) CD4^+^ T cells (CD3^+^CD4^+^) and b) CD8^+^ T cells (CD3^+^CD8^+^) in primary tumors of various groups of mice (gated on CD3^+^ cells). n = 3. Data are presented as mean ± SD. **P < 0.01.


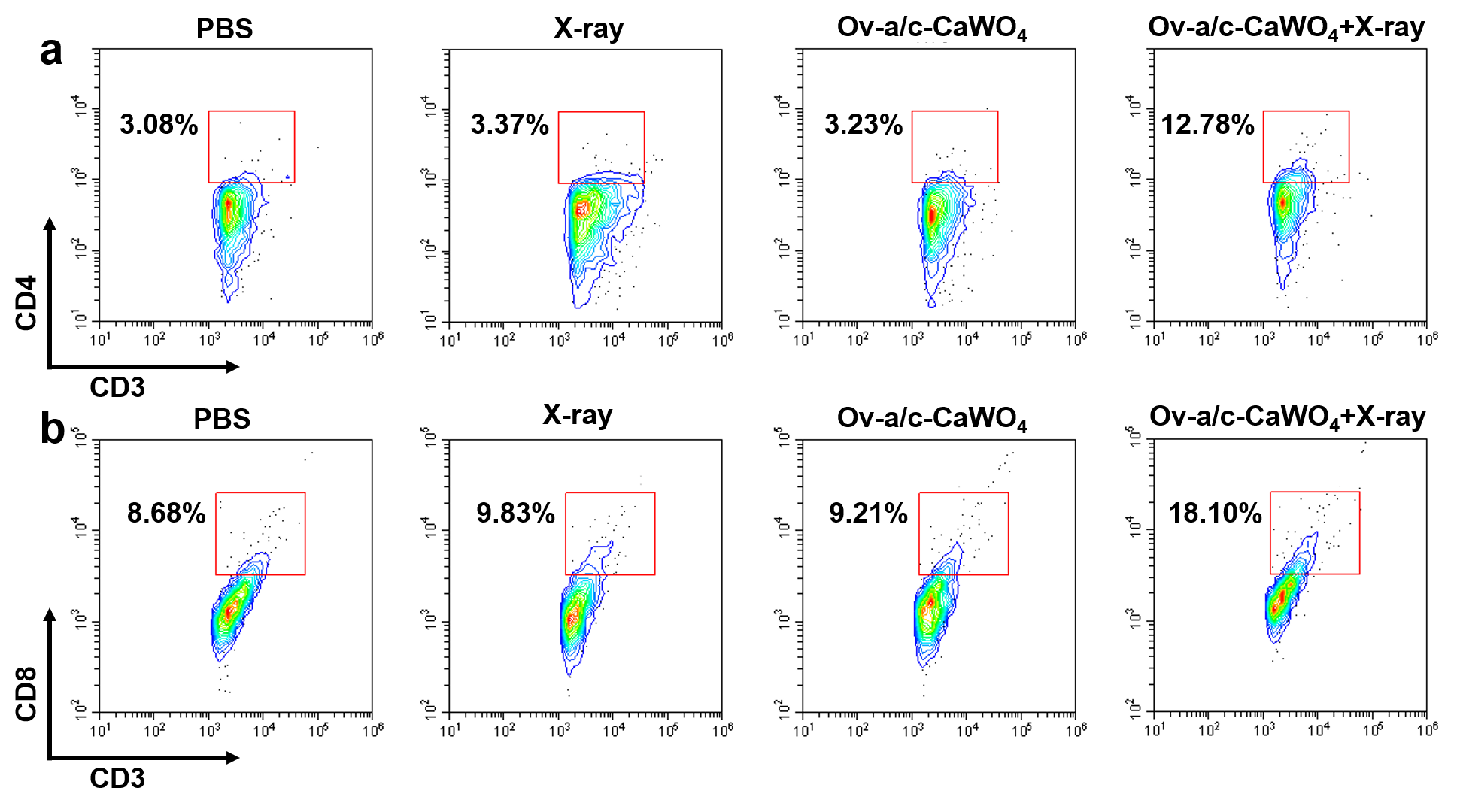


**Figure S31.** Flow cytometry analysis of a) CD4^+^ T cells (CD3^+^CD4^+^) and b) CD8^+^ T cells (CD3^+^CD8^+^) in distant tumors of bilateral 4T1 tumor-bearing mice after different treatments (gated on CD3^+^ cells).


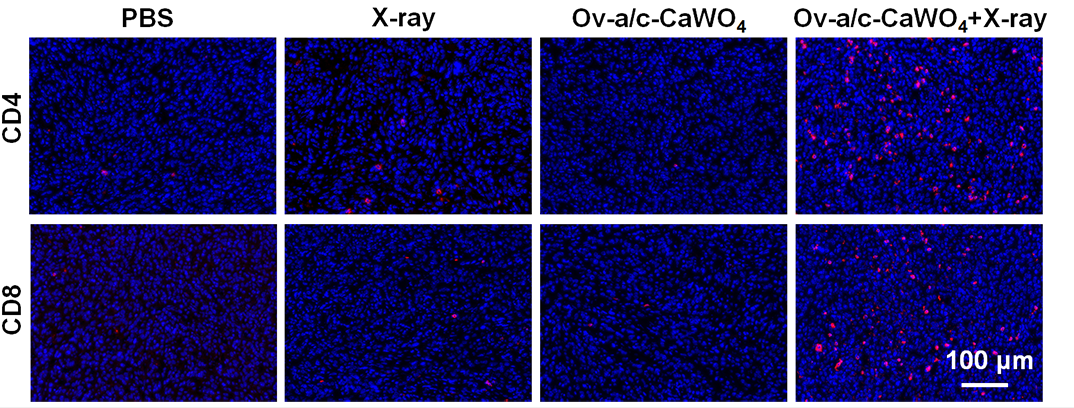


**Figure S32.** Representative immunofluorescence images of CD4^+^ and CD8^+^ T cells in primary tumors of different groups of mice.


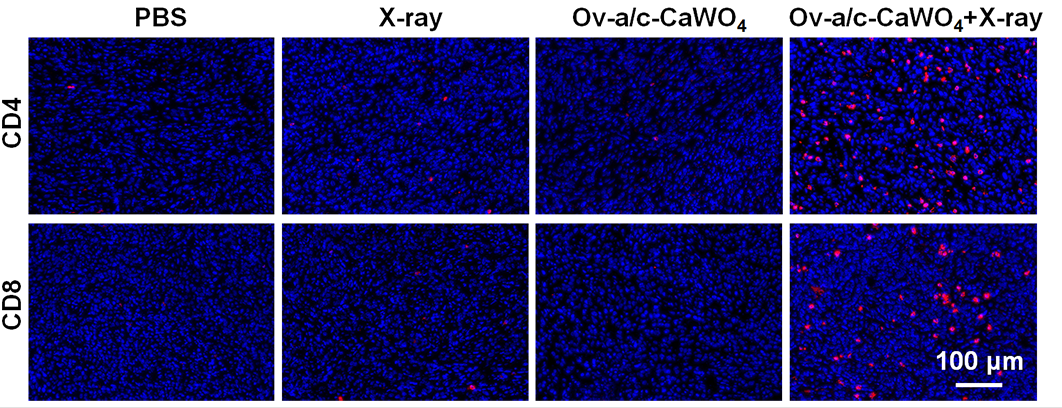


**Figure S33.** Representative immunofluorescence images of CD4^+^ and CD8^+^ T cells in distant tumors of different groups of mice.


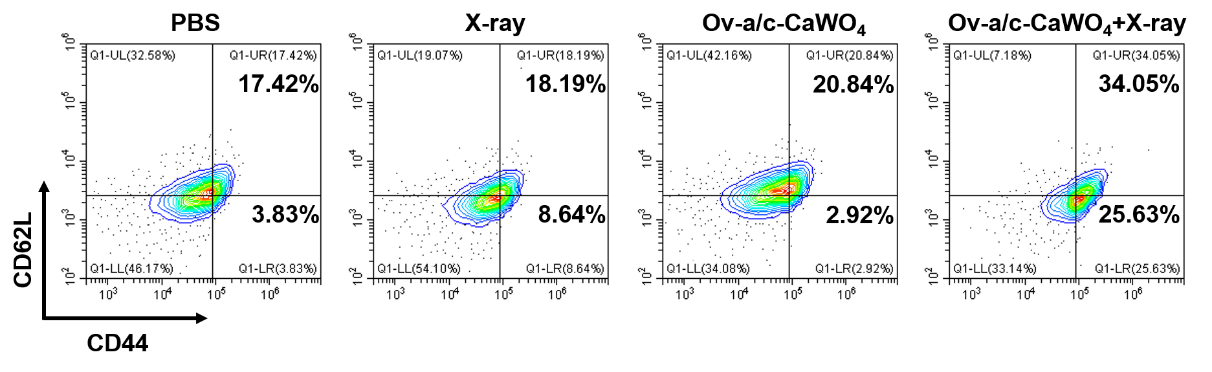


**Figure S34.** Flow cytometry analysis of effector memory T cells (CD3^+^CD8^+^CD44^+^CD62L^−^) and central memory T cells (CD3^+^CD8^+^CD44^+^CD62L^+^) in spleens of various groups of mice (gated on CD8^+^ T cells).
